# Rewiring carotenoid biosynthesis in plants using a viral vector

**DOI:** 10.1038/srep41645

**Published:** 2017-01-31

**Authors:** Eszter Majer, Briardo Llorente, Manuel Rodríguez-Concepción, José-Antonio Daròs

**Affiliations:** 1 Instituto de Biología Molecular y Celular de Plantas, Consejo Superior de Investigaciones Centíficas-Universidad Politécnica de Valencia, 46022 Valencia, Spain; 2 Centre for Research in Agricultural Genomics (CRAG) CSIC-IRTA-UAB-UB, Campus UAB Bellaterra, 08193 Barcelona, Spain

## Abstract

Plants can be engineered to sustainably produce compounds of nutritional, industrial or pharmaceutical relevance. This is, however, a challenging task as extensive regulation of biosynthetic pathways often hampers major metabolic changes. Here we describe the use of a viral vector derived from *Tobacco etch virus* to express a whole heterologous metabolic pathway that produces the health-promoting carotenoid lycopene in tobacco tissues. The pathway consisted in three enzymes from the soil bacteria *Pantoea ananatis*. Lycopene is present at undetectable levels in chloroplasts of non-infected leaves. In tissues infected with the viral vector, however, lycopene comprised approximately 10% of the total carotenoid content. Our research further showed that plant viruses that express *P. ananatis* phytoene synthase (crtB), one of the three enzymes of the heterologous pathway, trigger an accumulation of endogenous carotenoids, which together with a reduction in chlorophylls eventually result in a bright yellow pigmentation of infected tissues in various host-virus combinations. So, besides illustrating the potential of viral vectors for engineering complex metabolic pathways, we also show a yellow carotenoid-based reporter that can be used to visually track infection dynamics of plant viruses either alone or in combination with other visual markers.

Given their photoautotrophic nature and stunning biosynthetic capacity, plants represent exceptional systems from which to sustainably obtain valuable compounds. Much interest has been shown in engineering plants to improve their nutritional quality, to increase the yield of natural compounds, or to produce novel metabolites of industrial and pharmaceutical interest^1^. However, standard plant metabolic engineering typically relies on the generation of stable transgenic lines, which is a time-consuming and resource-intensive process. Development of novel metabolic engineering approaches, which are easy to implement should therefore be explored to overcome current limitations and to optimize production yields. In this context, virus-derived systems circumvent the need for stable plant transformation and enable the efficient simultaneous expression of heterologous genes on a timescale of a few days^2^,^3^,^4^,^5^,^6^.

In our previous work, we induced the biosynthesis of large amounts of plant anthocyanin pigments in tobacco (*Nicotiana tabacum* L.) leaves with a vector derived from *Tobacco etch virus* (TEV) that expressed the *Antirrhinum majus* transcription factors Rosea1 and Delila^7^,^8^,^9^. TEV is a cytoplasmic replicating plus-strand RNA virus that belongs to the genus *Potyvirus* in the family *Potyviridae*. Potyviruses are useful expression systems in plants for two main reasons. First, they can accommodate large amounts of extra genetic material in their elongated and flexuous capsids without losing infectivity^10^. Second, their expression strategy involves the release of different proteins from a large viral polyprotein by viral-encoded proteases. Insertion into the potyviral genome of heterologous sequences separated by the sequence motifs targeted by these proteases allows the production of encoded proteins in equimolar amounts^11^. In the TEV-derived expression system that we developed, the ca. 1.5 kb viral cistron that codes for the RNA-dependent RNA polymerase *nuclear inclusion b* (NIb) was replaced with a cassette that contains the heterologous sequences flanked by the native cleavage sites of the viral *nuclear inclusion a* protease (NIaPro). This strategy increases the space to accommodate extra genes while provided advantages from a biosafety viewpoint as the TEV-derived vectors can infect only plants in which NIb is supplied in *trans*^9^,^12^. Because the viral vector could accommodate several genes, we reasoned that it might serve to incorporate non-native metabolic pathways to plant cells. Here we test this possibility using a bacterial multistep pathway for the production of carotenoids.

Carotenoids are valuable for both health-promoting and economically-relevant reasons. Generally, animals cannot synthesize carotenoids but take them in their diets as a source of pigments and essential retinoids (including vitamin A, mainly produced from β-carotene). In humans, dietary carotenoids such as phytoene, lycopene and lutein have also been shown to act as health-promoting phytonutrients^13^,^14^. Carotenoids such as astaxanthin and canthaxanthin, which are not naturally produced by plants, are widely used in the chemical, pharmaceutical, cosmetic, feed and food industries, and thus represent really interesting targets for biotechnological production. All photosynthetic organisms and some non-photosynthetic bacteria and fungi synthesize carotenoids. In plants, C_40_ carotenoids are produced and stored in plastids (Fig. 1). As all isoprenoids, carotenoids derive from the basic C_5_ building units isopentenyl diphosphate (IPP) and dimethylallyl diphosphate (DMAPP). In the cytosol, these units are produced by the mevalonate (MVA) pathway and are then used mainly for the biosynthesis of sterols (Fig. 1). In plastids, however, IPP and DMAPP are produced by the completely unrelated methylerythritol 4-phosphate (MEP) pathway and used for the production of carotenoids and other photosynthesis-related compounds^15^. The condensation of three IPP molecules with one DMAPP acceptor generates C_20_ geranylgeranyl diphosphate (GGPP) in a reaction catalyzed by GGPP synthase (GGPPS). The first committed step of the carotenoid pathway, catalyzed by the enzyme phytoene synthase (PSY), produces phytoene from two GGPP molecules. Desaturation and isomerization reactions catalyzed by at least four enzymes in plants eventually transform the non-colored phytoene molecule into lycopene, a red carotenoid. Further modifications of lycopene subsequently generate β-carotene and derived xanthophylls in one branch of the pathway and lutein in the other branch (Fig. 1).

Carotenoid metabolic engineering in plants has focused on potentiating, modifying, removing or adding components to the endogenous plastid-localized MEP and carotenoid pathways^16^. Regrettably, engineering plant pathways toward high-level carotenoid biosynthesis has proven extremely challenging given the extensive multilevel regulatory constraints^17^,^18^,^19^. In the present work, we extend the use of viral vectors in plants to express a full heterologous pathway to synthesize lycopene from cytosolic (i.e. MVA-derived) isoprenoid precursors. As an indirect result of this research, we also describe a carotenoid-based yellow reporter, which can be very useful as a color-based system to track the infection dynamics of plant viruses. We demonstrate the excellent performance of this visual marker in a series of plant-virus combinations, including co-infection with a previously described anthocyanin-based red colored reporter^7^ to simultaneously follow the distribution of two different viral infections based on their clearly distinguishable associated colors.

## Results

### Redirecting cytosolic isoprenoid biosynthesis to produce lycopene in tobacco leaves infected with an engineered viral vector

**Figure 1:**
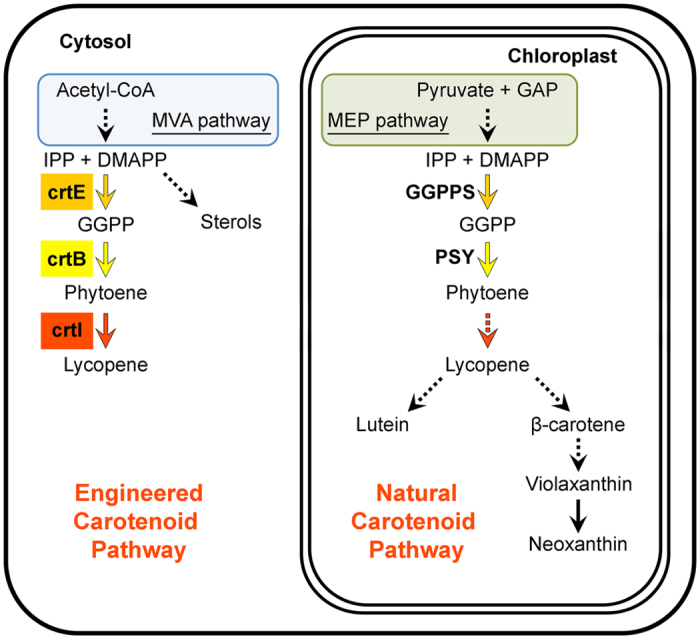
Schematic overview of the endogenous plant isoprenoid pathways and the virus-enabled metabolic engineering approach implemented in this study. The endogenous MEP and carotenoid pathways are localized in the chloroplast. The natural MVA pathway and the engineered carotenoid biosynthetic pathway are localized in the cytosol. Solid and dashed arrows represent single or multiple enzymatic steps, respectively. The steps catalyzed by *Pantoea ananatis* crtE, crtB and crtI are indicated. GAP, glyceraldehyde-3-phosphate; IPP, isopentenyl diphosphate; DMAPP, dimethylallyl diphosphate; GGPP, geranylgeranyl diphosphate; crtE/GGPPS, GGPP synthase; crtB/PSY, phytoene synthase; crtI, phytoene desaturase.

Using the TEV-based system that we previously developed^7^,^8^,^9^, we aimed to explore whether we could express a whole multistep heterologous metabolic pathway in plants. We chose to engineer a three-enzyme pathway from the soil bacterium *Pantoea ananatis*^20^ to synthesize the health-promoting carotenoid lycopene in the cytosol of tobacco leaf cells. In chloroplasts, lycopene is an intermediate of the carotenoid pathway that is readily converted into lutein, β-carotene and downstream xanthophylls (Fig. 1), and it normally goes undetected in tobacco leaves^21^,^22^,^23^,^24^. When produced and accumulated at high enough levels, however, it provides a distinctive red color (e.g. in ripe tomato and watermelon fruits). As cytosolic MVA-derived IPP and DMAPP are used mostly to synthesize C_15_ farnesyl diphosphate for the production of downstream isoprenoids such as sterols, we hypothesized that enhanced GGPP supply might be needed for extraplastidial carotenoid biosynthesis. We therefore incorporated the *P. ananatis crtE* gene that encodes GGPPS as the first step of the engineered pathway. The second gene of the pathway was *crtB*, which encodes PSY. The third selected gene, *crtI*, encodes the bacterial enzyme that directly converts phytoene into lycopene (Fig. 1).

**Figure 2:**
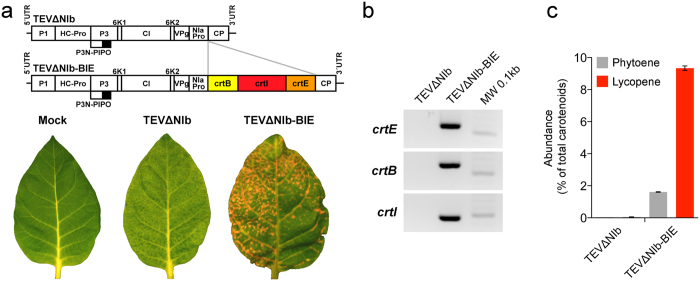
Production of lycopene in the cytosol of the virus-infected plant cells. (**a**) Schematic representation of TEV clones TEVΔNIb and TEVΔNIb-BIE. TEV cistrons P1, HC-Pro, P3, P3N-PIPO, 6K1, CI, 6K2, VPg, NIaPro and CP are represented by rectangles and the 5′ and 3′ untranslated regions (UTR) by black lines. The NIb cistron is deleted. *P. ananatis* crtB, crtI and crtE are represented by yellow, red and orange rectangles, respectively. Lower panel images correspond to representative systemic leaves of the tobacco plants mock-inoculated or infected with TEVΔNIb or TEVΔNIb-BIE. Pictures were taken at 10 dpi. (**b**) Analyses by RT-PCR and electrophoresis separation of the viral RNA that encode crtE, crtB and crtI from the symptomatic systemic leaves infected with TEVΔNIb or TEVΔNIb-BIE. (**c**) Accumulation of phytoene and lycopene in the tobacco systemic leaves infected with TEVΔNIb or TEVΔNIb-BIE. Metabolites were extracted from infected tissues and analyzed by HPLC. The average abundance relative to total carotenoid contents in n = 4 independent samples is represented. Error bars indicate SEM.

A cassette that contains the coding regions of *P. ananatis* genes *crtB, crtI* and *crtE* separated by artificial sequences that code for a peptide efficiently cleaved by NIaPro^9^, was inserted into the TEV vector to generate the viral clone TEVΔNIb-BIE (Fig. 2a and Supplementary Fig. S1). TEV replicates and expresses its proteins in the cytoplasm of infected plant cells. The heterologous *P. ananatis* proteins were not tagged with transit peptides to target the chloroplast. Nonetheless, to make sure that these bacterial proteins do not contain intrinsic signals to be targeted to the chloroplasts, we expressed green fluorescent protein (GFP)-tagged versions of the individual enzymes used in the synthetic pathway. A confocal laser scanning fluorescence microscopy analysis indicated that *P. ananatis* crtE-GFP and crtI-GFP are clearly excluded from chloroplasts (Supplementary Fig. S2). The crtB-GFP fusion produced much lower levels of fluorescence. While most of the signal was found in the cytoplasm, some fluorescence was also detected in chloroplasts (Supplementary Fig. S2). However, comparison with controls lacking GFP suggested that this plastidial signal might be due to overlapping with chlorophyll autofluorescence, suggesting that most (if not all) crtB-GFP is excluded from plastids. The recombinant TEVΔNIb-BIE was used to inoculate tobacco plants that constitutively express the viral NIb protein^12^. As a control, we used an empty viral vector that expressed no recombinant protein (TEVΔNIb; Fig. 2a and Supplementary Fig. S1). At 10 days post-inoculation (dpi), systemic (non-inoculated) leaves from the plants infected with TEVΔNIb-BIE exhibited spots and areas with a distinctly red coloration, suggestive of lycopene accumulation (Fig. 2a). RNA analysis via specifically designed primers and reverse transcription-polymerase chain reactions (RT-PCR) indicated that *crtE, crtB* and *crtI* were expressed in these tissues (Fig. 2b). More importantly, HPLC analysis revealed the presence of phytoene (the product of crtB) and relatively high lycopene levels in the leaf areas infected with TEVΔNIb-BIE, but not in those infected with the empty TEVΔNIb vector (Fig. 2c). Lycopene comprised approximately 10% of the total carotenoid content in the symptomatic red tissues of leaves infected with TEVΔNIb-BIE (Fig. 2c), reaching levels of ca. 150 μg per g of dry weight.

### TEV-mediated expression of crtB induces a characteristic yellow coloration in infected tissue

**Figure 3:**
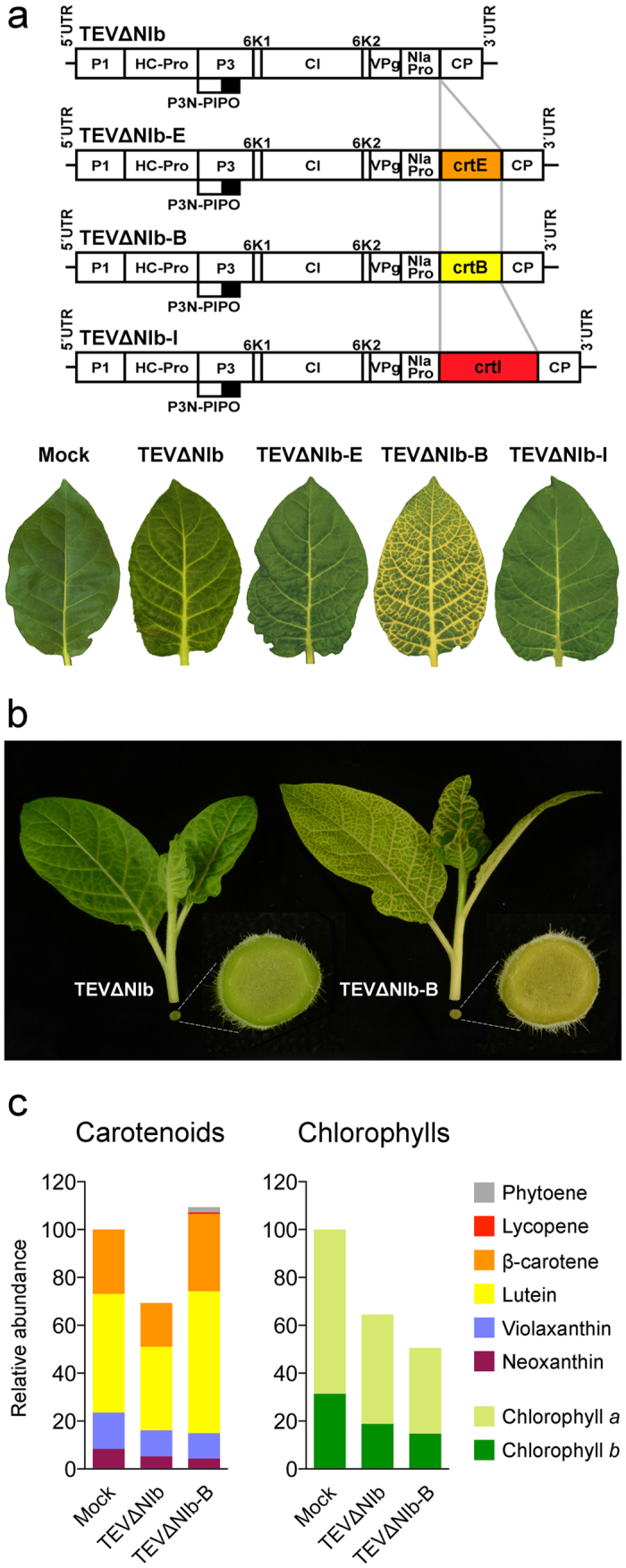
A yellow visual marker to track plant virus infection. (**a**) Schematic presentation of viral clones TEVΔNIb, TEVΔNIb-E, TEVΔNIb-B, and TEVΔNIb-I. Pictures in the lower part of the panel show systemic leaves of representative tobacco plants mock-inoculated or infected with TEVΔNIb, TEVΔNIb-E, TEVΔNIb-B, and TEVΔNIb-I. (**b**) Visual symptoms in systemic leaves and stems of tobacco plants infected with TEVΔNIb or TEVΔNIb-B. (**c**) Carotenoid and chlorophyll contents of tobacco leaves either infected with the indicated virus clones or treated with a mock solution. The average content in symptomatic systemic leaves from n = 4 independent plants at 10 dpi is represented.

To confirm that the observed production of lycopene required the complete pathway, we constructed three recombinant TEV clones in which each *P. ananatis* biosynthetic enzyme was expressed alone (Fig. 3a and Supplementary Fig. S1). The NIb-expressing tobacco plants were inoculated with these recombinant viruses, and a few dpi plants started to show typical symptoms of TEV infection. Strikingly, the tissues infected with TEVΔNIb-B exhibited a bright yellow coloration at 10 dpi while no difference in coloration was noted between the tissues infected with TEVΔNIb-E or TEVΔNIb-I and the control tissues infected with TEVΔNIb (Fig. 3a). Stems of the plants infected with TEVΔNIb-B also exhibited a distinctive yellow pigmentation that was not observed in the tissues infected with TEVΔNIb (Fig. 3b). This was unexpected because phytoene, the direct product of crtB activity (Fig. 1), is colorless. Interestingly, HPLC analysis of the carotenoid profile of the infected leaves showed that TEVΔNIb-B stimulated the accumulation of higher levels of chloroplastic (i.e. endogenous) carotenoids compared to empty vector control TEVΔNIb (Fig. 3c). In particular, the orange carotenoid β-carotene and the yellow carotenoid lutein (Fig. 1) were 70–75% more abundant in the symptomatic TEVΔNIb-B-infected yellow tissues (Fig. 3c). These tissues also presented an approximate 50% concomitant reduction in chlorophylls (Fig. 3c). A similar reduction in chlorophylls, but also in carotenoids, was observed when comparing the tissues infected with the empty TEVΔNIb vector to those from the mock-inoculated plants (Fig. 3c). This indicates that chloroplast functions are negatively impacted by viral infection. Collectively, these findings suggested that the yellow pigmentation that derived from the virus-mediated expression of crtB was likely caused by a combination of factors, including viral infection, which caused chlorosis due to a reduction in the levels of photosynthetic pigments, and crtB activity, which triggered increased carotenoid content.

**Figure 4:**
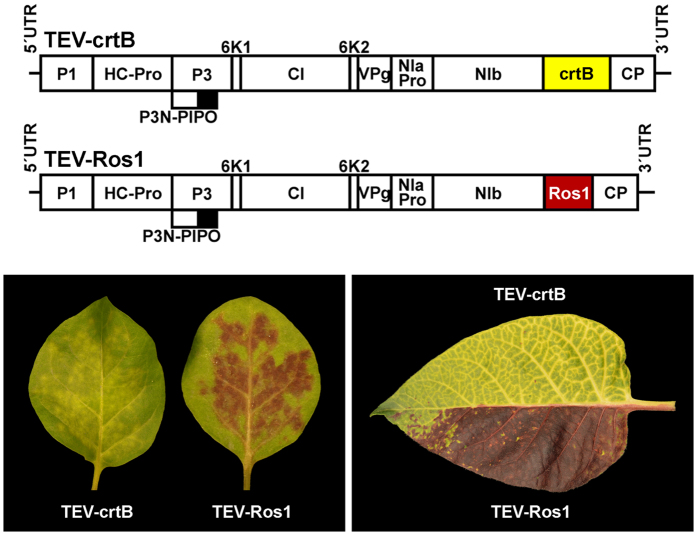
Simultaneous visual tracking of co-infecting plant viruses using crtB and Rosea1 markers. Schematic presentation of recombinant clones TEV-crtB and TEV-Ros1. The crtB and Rosea1 (Ros1) markers are represented with yellow and red boxes, respectively. Other details are as in Fig. 2a. The lower left picture shows tobacco leaves mechanically inoculated with TEV-crtB and TEV-Ros1 at 7 dpi. The lower right picture is a representative upper non-inoculated leaf co-infected with both recombinant viruses at 12 dpi.

Regardless of the molecular mechanism that caused the yellow pigmentation observed in the plants infected with TEVΔNIb-B, we reasoned that *P. ananatis* crtB could be used as a reporter marker to visually track virus infection in plants. We previously described how the use of the transcription factor Rosea1 from *A. majus* (an activator of anthocyanin biosynthesis) allowed to visually monitor infection dynamics in some plant-virus combinations based on the reddish color of the cells that accumulate anthocyanins^7^,^8^. To evaluate whether the simultaneous use of these two color-based visual markers could actually serve to monitor the infection of two different virus populations, we constructed a new TEV-crtB clone by inserting the sequence coding for crtB between the NIb and CP (coat protein) cistrons in the TEV genome, in exactly the same position as the *Rosea1* gene in the TEV-Ros1 clone (Fig. 4 and Supplementary Fig. S3)^7^,^8^. Note that, unlike the above-described TEVΔNIb-B, the TEV-crtB construct contains the full-length TEV genome, including NIb. As in TEV-Ros1, the *crtB* gene is also flanked by artificial sequences to promote its proteolytic processing from the viral polyprotein (Supplementary Fig. S3). Leaves of wild-type tobacco plants were inoculated with both TEV-crtB and TEV-Ros1, with each recombinant virus in one half of the same leaf. After 4 days, plants started to show symptoms of infection and the symptomatic tissues became pigmented approximately two days later. Depending on which recombinant virus (TEV-crtB or TEV-Ros1) invaded the tissue, both inoculated and systemic symptomatic tissues showed alternative yellow or red coloration (Fig. 4). Yellow and red pigmentation nicely marked the particular tissue sections colonized by different viruses in systemic leaves at 12 dpi (Fig. 4).

### A new wide-spectrum visual marker to track the dynamics of virus infection in plants

**Figure 5:**
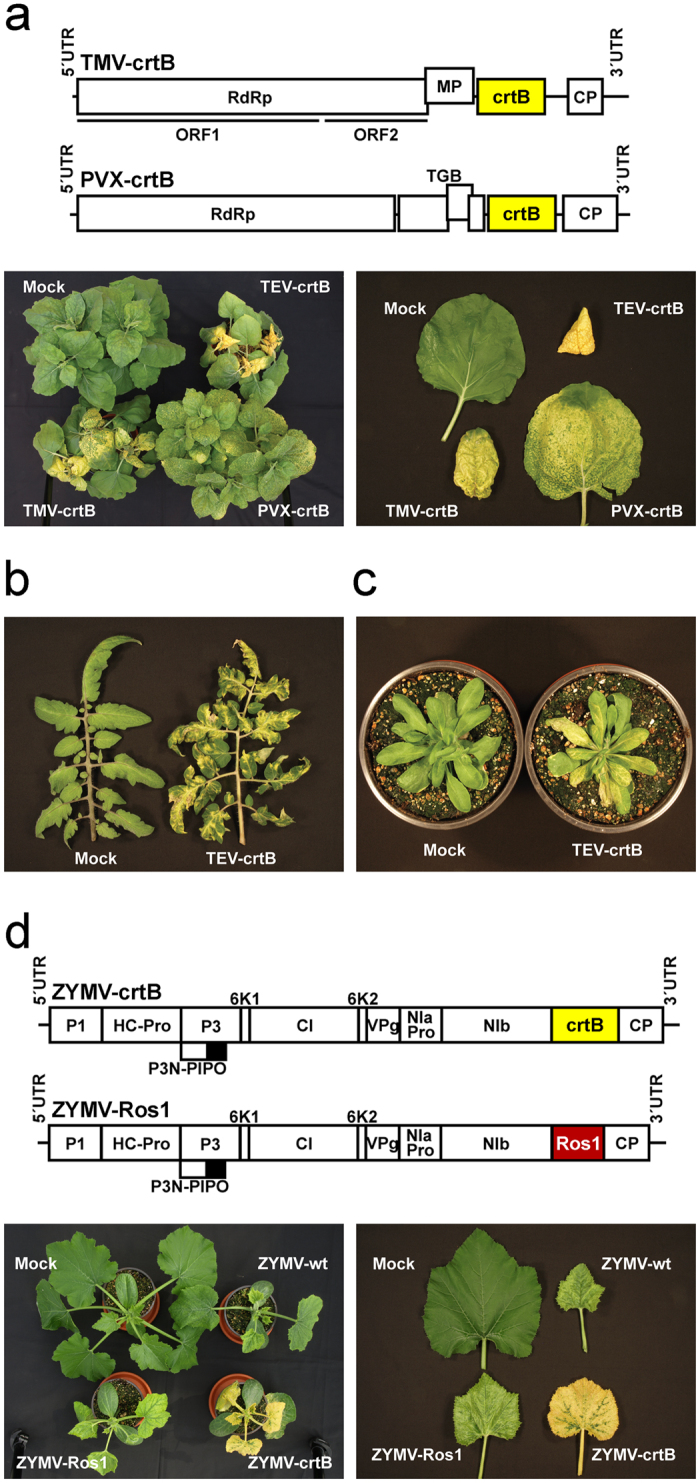
Performance of the crtB marker in different virus and plant species. (**a**) Schematic presentation of recombinant clones TMV-crtB and PVX-crtB. RNA-dependent RNA polymerase (RdRp), movement protein (MP), coat protein (CP) and triple gene block (TGB) proteins are represented by open boxes. 5′ and 3′ UTRs are represented by lines. crtB is denoted by a yellow box. The pictures in the lower part of the panel show *N. benthamiana* plants (left) and representative leaves from these plants (right) mock-inoculated or inoculated with TEV-crtB, TMV-crtB or PVX-crtB at 15 dpi. (**b**) Leaves from tomato plants mock-inoculated or infected with TEV-crtB at 15 dpi. (**c**) *A. thaliana* plants mock-inoculated or infected with TEV-crtB at 15 dpi. (**d**) Schematic presentation of the recombinant ZYMV clones tagged with crtB or Rosea1. Details are as in Figs 2a and 4a. Pictures in the lower part of the panel show representative zucchini plants (left) and leaves from these plants (right) mock-inoculated or infectd with wild-type ZYMV (ZYMV-wt), ZYMV-Ros1 or ZYMV-crtB at 11 dpi.

To test whether *P. ananatis* crtB could be used as a general marker in different plant-virus combinations, we constructed recombinant clones of two viruses that belong to different families: *Tobacco mosaic virus* (TMV; family *Virgaviridae*) and *Potato virus X* (PVX; family *Flexiviridae*), both of which harbored the crtB marker. The resulting clones, TMV-crtB and PVX-crtB (Fig. 5a and Supplementary Fig. S3), as well as TEV-crtB, were used to inoculate *Nicotiana benthamiana* plants. A few days later, plants developed symptoms of infection and the symptomatic tissues subsequently turned yellow (Fig. 5a). The intensity and extension of yellow pigmentation differed according to virus, which indicate that each species has its particular infection dynamics in *N. benthamiana*. We also analyzed whether the crtB marker was useful for tracking virus infection in an important crop like tomato (*Solanum lycopersicum* L.) and in the model plant *Arabidopsis thaliana*. After inoculation with TEV-crtB, the symptomatic tissues of both the tomato (Fig. 5b) and *A. thaliana* (Fig. 5c) plants exhibited yellow coloration clearly discernible to the naked eye. Finally, we also analyzed the behavior of the new marker in cucurbitaceous plants, a family that includes many important crops. Our own previous observations have indicated that the Rosea1 marker does not function in this family^25^. To test whether the crtB marker could be extended to track virus infection in cucurbits, we constructed two recombinant clones of *Zucchini yellow mosaic virus* (ZYMV; genus *Potyvirus*, family *Potyviridae*) that harbor the sequences, which encode either crtB (ZYMV-crtB) or Rosea1 (ZYMV-Ros1) (Fig. 5d and Supplementary Fig. S3). Zucchini (*Cucurbita pepo* L.) plantlets were inoculated with both recombinant viruses, and also with an untagged virus control. Some days later, all the inoculated plants developed symptoms of infection, but marker specific coloration was observed only for ZYMV-crtB (Fig. 5d).

## Discussion

Engineering plant metabolism to produce compounds of nutritional, industrial, or pharmaceutical relevance remains an important goal given the economic benefit of using sunlight to fuel sustainable production methods. However, this is currently a challenging process that is often hampered by the highly complex regulation of plant metabolism. Orthogonal systems, which are mechanistically and spatially separated from those hardwired by evolution and tightly regulated by the host, can potentially operate with improved performance. The feasibility and efficiency of our orthogonal approach was confirmed by the overproduction of lycopene in tobacco leaf cells (Fig. 2). Heterologous carotenoid pathways from bacterial origin have been successfully introduced in crop plants using stable transformation^26^,^27^,^28^. In addition, previous metabolic engineering efforts showed that precursors from the cytosolic MVA pathway can be redirected to synthesize isoprenoids normally produced in plastids, such as monoterpenes^29^,^30^. However, the production of carotenoids outside plastids had not been yet attempted in plants. The accumulated amount of lycopene in infected tobacco leaves (ca. 150 μg per g of dry weight) is similar to that obtained in yeast (*Saccharomyces cerevisiae*) cells engineered to produce lycopene from the endogenous MVA pathway using the same *P. ananatis* crtE, crtB and crtI enzymes^31^. Recent optimizations in yeast increased the production of lycopene 5 to 10-fold^32^,^33^, reaching levels similar to those found in the specialized plastids (chromoplasts) of red tomatoes^30^. In our experiments, the red tissues that accumulated lycopene soon became necrotized, possibly because lycopene is not normally produced in the plant cell cytosol, and overaccumulation of such a lipophilic compound, or its association with cell membranes, may cause undesired effects on cell functions. In any case, the transient expression and localized distribution of the viral vector used did not compromise plant survival, as carotenoid production was restricted to infected leaves. The reported system could be optimized (e.g. by creating new membrane structures to act as a deposit sink for carotenoids) and adapted to the production of carotenoids which do not naturally occur in plants, including astaxanthin and canthaxanthin, or other high-value isoprenoids. It is expected that optimization of this virus-based system could eventually improve both the production and the storage of the isoprenoid of interest in infected leaf cells while decreasing the undesired necrotic effect.

The fact that the TEV-mediated cytosolic expression of crtB turned infected tissue into a bright yellow color (Fig. 3) was an unexpected observation. Virus-infected plants show strong metabolic alterations and typically display symptoms such as chlorosis and necrosis. In particular, chloroplasts and photosynthesis have been widely recognized as common targets by many plant viruses^34^, and eventually result in low levels of photosynthetic pigments (chlorophylls and carotenoids), as we observed in the tissues infected with the empty vector (TEVΔNIb) (Fig. 3). The increased production of chloroplast carotenoids observed in tissues infected with crtB-producing viruses, however, is more complex to explain. Oxidative breakage of carotenoids into signaling molecules by carotenoid cleavage dioxygenase (CCD) enzymes located in the cytoplasm is critical for the control of carotenoid accumulation^19^. It is therefore possible that CCD-mediated or even non-enzymatic cleavage of crtB-produced phytoene might release an unknown signal triggering enhanced accumulation of chloroplast carotenoids. Alternatively, some crtB molecules might enter the chloroplasts and produce phytoene to feed the endogenous carotenoid pathway. Further experimental insights will be required in order to unveil the exact underlying mechanism. In any case, the tissue pigmentation caused by virus-mediated crtB expression aroused a second, and initially unanticipated, goal of the research herein; that is, its use as a visual marker to track infection dynamics of plant viruses. Tracking plant viruses with reporter genes has contributed significantly to our understanding of the infection process, and has helped to decipher the role of host factors and to screen resistance genes. Most current reporter marker systems used in plant virology rely on fluorescent proteins^35^, which require specialized instrumentation for detection purposes. A visual marker, unlike fluorescent proteins, facilitates high-throughput screenings and allows continuous monitoring of the infectious process^7^,^8^. Previous reports showed that TMV-mediated expression of tomato PSY or pepper capsanthin-capsorubin synthase induced yellow pigmentation in infected tissues, although these plant enzymes were expressed including their corresponding chloroplast targeting peptides. Authors recognized the utility of these visual markers in the analysis of viral insert stability, replication and movement^36^,^37^. We previously demonstrated the effectiveness of plant metabolism-based reporter systems. Activating the biosynthesis of anthocyanins with the *A. majus* transcription factor Rosea1 has well revealed the infected tissues with a reddish coloration in many plant-virus combinations^7^,^8^. Pigment accumulation is cell-autonomous and occurs only in those tissues where the virus replicates. This metabolism-based visual marker is also quantitative because the amount of anthocyanin pigments correlates with viral load. The addition of a second reporter gene that functions upon the utilization of a different metabolic pathway and displays an alternative output color clearly extends the experimental possibilities of the visual system, and allows the infection dynamics of two different viruses or of two different strains of the same virus in co-infected plants to be tracked (Fig. 4). Our results indicate crtB to be a very versatile marker that is capable of inducing yellow coloration when used with several viruses of different genus and families, including TEV, TMV, PVX, and ZYMV (Fig. 5). Most interestingly, it works in a wide variety of host species, including the model plants *A. thaliana* and tomato (Fig. 5). Our experiments with cucurbitaceous plants (Fig. 5d) showed that crtB may be a more general marker than Rosea1 because of the ubiquitous presence of the carotenoid pathway in plants.

In summary, we herein engineered a heterologous complete pathway in an easy-to-manipulate viral vector and rewired the plant cell metabolism for extraplastidial lycopene production, which represents a new way for future biotechnological approaches. For instance, we envisage that current plastid-based approach used for carotenoid biofortification in crops^38^ could be further complemented by extraplastidial carotenoid biosynthesis, thus expanding our capacity to develop carotenoid-enriched food for improving human nutrition and health. Additionally, we describe the discovery of crtB as an excellent wide-spectrum yellow visual marker for tracking plant virus infections by impacting chloroplast metabolism, and we show that it can be combined with the red Rosea1 marker in experiments with two different output colors. Finally, our study joins others in demonstrating the enormous potential of viral vectors for engineering metabolic pathways in plants^2^,^9^,^36^,^37^,^39^,^40^,^41^,^42^,^43^,^44^. Further advances should combine the use of virus-derived vectors with stable genetic transformation, and even with the remodeling of the subcellular architecture in increasingly sophisticated engineering approaches to overaccumulate compounds of nutritional or economic interest in a cost-effective and scalable manner.

## Methods

### Plasmid clones

Plasmid pGTEVa^7^ contains the cDNA of an infectious wild-type TEV (TEV-wt), with GenBank accession number DQ986288, but including two silent and neutral mutations, G273A and A1119G, flanked by *Cauliflower mosaic virus* (CaMV) 35S promoter and terminator in a binary vector that derives from pCLEAN-G181^45^. This plasmid was used as a parental to construct plasmids with TEV clones TEVΔNIb, TEVΔNIb-BIE, TEVΔNIb-E, TEVΔNIb-B, TEVΔNIb-I, by standard molecular biology techniques, including PCR amplification with high-fidelity Phusion DNA polymerase (Thermo Scientific); DNA assembly by digestion with type-IIS restriction enzyme BsaI-HF (New England Biolabs), followed by ligation with T4 DNA ligase (Thermo Scientific)^46^; and Gibson DNA assembly^47^ with the NEBuilder HiFi DNA Assembly Master Mix (New England Biolabs). *P. ananatis crtE, crtB* and *crtI* were amplified from plasmid pACCRT-EIB^48^. The sequences of TEVΔNIb and all the recombinant TEVΔNIb-derived clones are seen in Supplementary Fig. S1 (see Supplementary Material). A plasmid with recombinant clone TEV-crtB (Supplementary Fig. S3) was also constructed from parental plasmid pGTEVa. Viral clone TEV-Ros1, which includes *A. majus* Rosea1 between the NIb and CP cistron (Supplementary Fig. S3), was contained in the previously described plasmid pGTEV-Ros1(NIb/CP)^7^. Viral clone TMV-crtB (Supplementary Fig. S3) derives from the TMV 30B expression vector, which includes the 3′ end of *Tobacco mild green mosaic virus*^49^. It was constructed in a binary plasmid that derived from pCLEAN-G181, including the CaMV 35S promoter and terminator and a ribozyme to produce the 3′ end of the viral RNA that derived from that of the minus strand of the human *Hepatitis delta virus*^50^. Viral clone PVX-crtB (Supplementary Fig. S3) was constructed by starting from pgR107^51^, but recombinant crtB was expressed from a heterologous promoter that derived from *Bamboo mosaic virus* and contained the deletion of the 29 initial codons of PVX CP^52^. Plasmid pGZYMV, which contained a ZYMV (Supplementary Fig. S2) infectious clone (GenBank accession number KX499498), was also a derivative of pCLEAN-G181, including the CaMV 35S promoter and terminator. This plasmid was used to construct recombinant clones ZYMV-crtB and ZYMV-Ros1 (Supplementary Fig. S2). For the transient expression in *N. benthamiana* leaves, full-length *crtE, crtB* and *crtI* sequences were amplified from pACCRT-EIB and cloned into plasmid pDONR207 by the Gateway technology (Invitrogen). Sequences were then subcloned into plasmid pGWB405 to obtain fusions with a carboxy-terminal GFP tag^53^. The sequences of all the plasmids were confirmed by standard DNA sequencing techniques.

### Plant inoculation

The plasmids that contained the different viral clones were electroporated into *Agrobacterium tumefaciens* C58C1, which carried helper plasmid pCLEAN-S48^45^. The cultures of transformed *A. tumefaciens* were used to agroinoculate *N. benthamiana* plants^54^ that were cultivated in a growth chamber at 25 °C with a 12-h photoperiod. As soon as symptoms were detected in systemic tissue, aliquots of symptomatic tissue were collected and stored frozen at −80 °C. The crude extracts from these aliquots were used to mechanically inoculate 4-week-old *N. tabacum* cv. Xanthi nc, 5-week-old *N. benthamiana*, 4-week-old *A. thaliana* ecotype Landsberg erecta, 3-week-old tomato cv. Marglobe and 12-day-old zucchini cv. MU-CU-16 plants, as previously described^54^. The transient expression in the *N. benthamiana* leaves was performed as described elsewhere^55^.

### Analysis of metabolites in infected tissues

Carotenoids and chlorophylls were extracted from 4 mg of lyophilized tobacco leaf tissue using 1 ml cold extraction solvent, and were analyzed by HPLC in an Agilent 1200 series HPLC system (Agilent Technologies), as previously described^56^.

### RNA analysis

Leaf samples of the symptomatic tissue infected with TEVΔNIb or TEVΔNIb-BIE were harvested and total RNA was extracted with the Maxwell 16 LEV simplyRNA Tissue Kit (Promega) according to the manufacturer’s instructions. Purified RNAs were quantified by spectroscopy in a NanoDrop apparatus (Thermo Scientific) and RNA integrity was evaluated by agarose gel electrophoresis. The First Strand cDNA Synthesis Kit (Roche) was used to generate cDNA according to the manufacturer’s instructions, with random primers and 1 μg of total RNA. The PCR reactions for *crtE, crtB* and *crtI* detection were performed with the Go Taq Green Master Mix (Promega) and primers Pa.crtE-F (5′-AACTGCTGGACGATTTGACC-3′); Pa.crtE-R (5′-CTCGGGCCTAACAGATTGAC-3′); Pa.crtB-F (5′-CTACGGCGAAGCAGGTTTAC-3′); Pa.crtB-R (5′-GCAGCAGCGTTAATTTTTCG-3′); Pa.crtI-F (5′-ATTACATGCCTGGCTTACGG-3′); and Pa.crtI-R (5′-GCTCCACAGAAAAGGCTGAG-3′). PCR products were analyzed by electrophoresis on 3% agarose gels and stained with ethidium bromide.

## Additional Information

**How to cite this article**: Majer, E. *et al*. Rewiring carotenoid biosynthesis in plants using a viral vector. *Sci. Rep.* **7**, 41645; doi: 10.1038/srep41645 (2017).

**Publisher's note:** Springer Nature remains neutral with regard to jurisdictional claims in published maps and institutional affiliations.

## Supplementary Material

Supplementary Information
