# Supplementary material for: Rewiring carotenoid biosynthesis in plants using a viral vector
Source: Sci Rep. 2017 Jan 31;7:41645. doi: 10.1038/srep41645 (PMC5282570; doi:10.1038/srep41645)
Supplement: Supplementary Information [file srep41645-s1.pdf]

SUPPLEMENTARY INFORMATION

Rewiring carotenoid biosynthesis in plants using a viral vector

Eszter Majer[1], Briardo Llorente[2], Manuel Rodríguez-Concepción[2] & José-Antonio Daròs[1]

[1]Instituto de Biología Molecular y Celular de Plantas, Consejo Superior de Investigaciones Centíficas-Universidad Politécnica de Valencia, 46022 Valencia, Spain. [2]Centre for Research in Agricultural Genomics (CRAG) CSIC-IRTA-UAB-UB, Campus UAB Bellaterra, 08193 Barcelona, Spain. Correspondence and requests for materials should be addressed to J.-A.D (email: jadaros@ibmcp.upv.es)

Figure S1. Full sequence of the viral vector TEVΔNIb and their derived recombinant clones TEVΔNIb-BIE, TEVΔNIb-E, TEVΔNIb-B and TEVΔNIb-I. TEV sequence corresponds to Genbank accession number DQ986288 including two silent mutations (G273A and A1119G, in red). Limits between TEV cistrons are marked on blue background. cDNAs corresponding to *P. ananatis crtB*, *crtI* and *crtE* are on yellow, red and olive backgrounds, respectively. In the inserted cDNAs, sequences corresponding to native and artificial TEV NIaPro cleavage sites are in black or blue, respectively.

>TEVΔNIb
AAAATAACAAATCTCAACACAACATATACAAAACAAACGAATCTCAAGCAATCAAGCATTCTACTTCTATTGCAG
CAATTTAAATCATTTCTTTTAAAGCAAAAGCAATTTTCTGAAAATTTTCACCATTTACGAACGATAGCCATGGCA
CTCATCTTTGGCACAGTCAACGCTAACATCCTGAAGGAAGTGTTCGGTGGAGCTCGTATGGCTTGCGTTACCAGC
GCACATATGGCTGGAGCGAATGGAAGCATTTTGAAGAAGGCAGAAGAAACCTCTCGTGCAATCATGCACAAACCA
GTGATCTTCGGAGAAGACTACATTACCGAGGCAGACTTGCCTTACACACCACTCCATTTAGAGGTCGATGCTGAA
ATGGAGCGGATGTATTATCTTGGTCGTCGCGCGCTCACCCATGGCAAGAGACGCAAAGTTTCTGTGAATAACAAG
AGGAACAGGAGAAGGAAAGTGGCCAAAACGTACGTGGGGCGTGATTCCATTGTTGAGAAGATTGTAGTGCCCCAC
ACCGAGAGAAAGGTTGATACCACAGCAGCAGTGGAAGACATTTGCAATGAAGCTACCACTCAACTTGTGCATAAT
AGTATGCCAAAGCGTAAGAAGCAGAAAAACTTCTTGCCCGCCACTTCACTAAGTAACGTGTATGCCCAAACTTGG
AGCATAGTGCGCAAACGCCATATGCAGGTGGAGATCATTAGCAAGAAGAGCGTCCGAGCGAGGGTCAAGAGATTT
GAGGGCTCGGTGCAATTGTTCGCAAGTGTGCGTCACATGTATGGCGAGAGGAAAAGGGTGGACTTACGTATTGAC
AACTGGCAGCAAGAGACACTTCTAGACCTTGCTAAAAGATTTAAGAATGAGAGAGTGGATCAATCGAAGCTCACT
TTTGGTTCAAGTGGCCTAGTTTTGAGGCAAGGCTCGTACGGACCTGCGCATTGGTATCGACATGGTATGTTCATT
GTACGCGGTCGGTCGGATGGGATGTTGGTGGATGCTCGTGCGAAGGTAACGTTCGCTGTTTGTCACTCAATGACA
CATTATAGCGACAAATCAATCTCTGAGGCATTCTTCATACCATACTCTAAGAAATTCTTGGAGTTGAGGCCAGAT
GGAATCTCCCATGAGTGTACAAGAGGAGTATCAGTTGAGCGGTGCGGTGAGGTGGCTGCAATCCTGACACAAGCA
CTTTCACCGTGTGGTAAGATCACATGCAAACGTTGCATGGTTGAAACACCTGACATTGTTGAGGGTGAGTCGGGA
GACAGTGTCACCAACCAAGGTAAGCTCCTAGCAATGCTGAAAGAACAGTATCCAGATTTCCCAATGGCCGAGAAA
CTACTCACAAGGTTTTTGCAACAGAAATCACTAGTAAATACAAATTTGACAGCCTGCGTGAGCGTCAAACAACTC
ATTGGTGACCGCAAACAAGCTCCATTCACACACGTACTGGCTGTCAGCGAAATTCTGTTTAAAGGCAATAAACTA
ACAGGGGCCGATCTCGAAGAGGCAAGCACACATATGCTTGAAATAGCAAGGTTCTTGAACAATCGCACTGAAAAT
ATGCGCATTGGCCACCTTGGTTCTTTCAGAAATAAAATCTCATCGAAGGCCCATGTGAATAACGCACTCATGTGT
GATAATCAACTTGATCAGAATGGGAATTTTATTTGGGGACTAAGGGGTGCACACGCAAAGAGGTTTCTTAAAGGA
TTTTTCACTGAGATTGACCCAAATGAAGGATACGATAAGTATGTTATCAGGAAACATATCAGGGGTAGCAGAAAG
CTAGCAATTGGCAATTTGATAATGTCAACTGACTTCCAGACGCTCAGGCAACAAATTCAAGGCGAAACTATTGAG
CGTAAAGAAATTGGGAATCACTGCATTTCAATGCGGAATGGTAATTACGTGTACCCATGTTGTTGTGTTACTCTT
GAAGATGGTAAGGCTCAATATTCGGATCTAAAGCATCCAACGAAGAGACATCTGGTCATTGGCAACTCTGGCGAT
TCAAAGTACCTAGACCTTCCAGTTCTCAATGAAGAGAAAATGTATATAGCTAATGAAGGTTATTGCTACATGAAC
ATTTTCTTTGCTCTACTAGTGAATGTCAAGGAAGAGGATGCAAAGGACTTCACCAAGTTTATAAGGGACACAATT
GTTCCAAAGCTTGGAGCGTGGCCAACAATGCAAGATGTTGCAACTGCATGCTACTTACTTTCCATTCTTTACCCA
GATGTCCTGAGTGCTGAATTACCCAGAATTTTGGTTGATCATGACAACAAAACAATGCATGTTTTGGATTCGTAT
GGGTCTAGAACGACAGGATACCACATGTTGAAAATGAACACAACATCCCAGCTAATTGAATTCGTTCATTCAGGT
TTGGAATCCGAAATGAAAACTTACAATGTTGGAGGGATGAACCGAGATATGGTCACACAAGGTGCAATTGAGATG
TTGATCAAGTCCATATACAAACCACATCTCATGAAGCAGTTACTTGAGGAGGAGCCATACATAATTGTCCTGGCA
ATAGTCTCCCCTTCAATTTTAATTGCCATGTACAACTCTGGAACTTTTGAGCAGGCGTTACAAATGTGGTTGCCA
AATACAATGAGGTTAGCTAACCTCGCTGCCATCTTGTCAGCCTTGGCGCAAAAGTTAACTTTGGCAGACTTGTTC
GTCCAGCAGCGTAATTTGATTAATGAGTATGCGCAGGTAATTTTGGACAATCTGATTGACGGTGTCAGGGTTAAC
CATTCGCTATCCCTAGCAATGGAAATTGTTACTATTAAGCTGGCCACCCAAGAGATGGACATGGCGTTGAGGGAA
GGTGGCTATGCTGTGACCTCTGAAAAGGTGCATGAAATGTTGGAAAAAAACTATGTAAAGGCTTTGAAGGATGCA
TGGGACGAATTAACTTGGTTGGAAAAATTCTCCGCAATCAGGCATTCAAGAAAGCTCTTGAAATTTGGGCGAAAG
CCTTTAATCATGAAAAACACCGTAGATTGCGGCGGACATATAGACTTGTCTGTGAAATCGCTTTTCAAGTTCCAC
TTGGAACTCCTGAAGGGAACCATCTCAAGAGCCGTAAATGGTGGTGCAAGAAAGGTAAGAGTAGCGAAGAATGCC
ATGACAAAAGGGGTTTTTCTCAAAATCTACAGCATGCTTCCTGACGTCTACAAGTTTATCACAGTCTCGAGTGTC
CTTTCCTTGTTGTTGACATTCTTATTTCAAATTGACTGCATGATAAGGGCACACCGAGAGGCGAAGGTTGCTGCA
CAGTTGCAGAAAGAGAGCGAGTGGGACAATATCATCAATAGAACTTTCCAGTATTCTAAGCTTGAAAATCCTATT
GGCTATCGCTCTACAGCGGAGGAAAGACTCCAATCAGAACACCCCGAGGCTTTCGAGTACTACAAGTTTTGCATT

GGAAAGGAAGACCTCGTTGAACAGGCAAAACAACCGGAGATAGCATACTTTGAAAAGATTATAGCTTTCATCACA
CTTGTATTAATGGCTTTTGACGCTGAGCGGAGTGATGGAGTGTTCAAGATACTCAATAAGTTCAAAGGAATACTG
AGCTCAACGGAGAGGGAGATCATCTACACGCAGAGTTTGGATGATTACGTTACAACCTTTGATGACAATATGACA
ATCAACCTCGAGTTGAATATGGATGAACTCCACAAGACGAGCCTTCCTGGAGTCACTTTTAAGCAATGGTGGAAC
AACCAAATCAGCCGAGGCAACGTGAAGCCACATTATAGAACTGAGGGGCACTTCATGGAGTTTACCAGAGATACT
GCGGCATCGGTTGCCAGCGAGATATCACACTCACCCGCAAGAGATTTTCTTGTGAGAGGTGCTGTTGGATCTGGA
AAATCCACAGGACTTCCATACCATTTATCAAAGAGAGGGAGAGTGTTAATGCTTGAGCCTACCAGACCACTCACA
GATAACGTGCACAAGCAACTGAGAAGTGAACCATTTAACTGCTTCCCAACTTTGAGGATGAGAGGGAAGTCAACT
TTTGGGTCATCACCGATTACAGTCATGACTAGTGGATTCGCTTTACACCATTTTGCACGAAACATAGCTGAGGTA
AAAACATACGATTTTGTCATAATTGATGAATGTCATGTGAATGATGCTTCTGCTATAGCGTTTAGGAATCTACTG
TTTGAACATGAATTTGAAGGAAAAGTCCTCAAAGTGTCAGCCACACCACCAGGTAGAGAAGTTGAATTCACAACT
CAGTTTCCCGTGAAACTCAAGATAGAAGAGGCTCTTAGCTTTCAGGAATTTGTAAGTTTACAAGGGACAGGTGCC
AACGCCGATGTGATTAGTTGTGGCGACAACATACTAGTATATGTTGCTAGCTACAATGATGTTGATAGTCTTGGC
AAGCTCCTTGTGCAAAAGGGATACAAAGTGTCGAAGATTGATGGAAGAACAATGAAGAGTGGAGGAACTGAAATA
ATCACTGAAGGTACTTCAGTGAAAAAGCATTTCATAGTCGCAACTAATATTATTGAGAATGGTGTAACCATTGAC
ATTGATGTAGTTGTGGATTTTGGGACTAAGGTTGTACCAGTTTTGGATGTGGACAATAGAGCGGTGCAGTACAAC
AAAACTGTGGTGAGTTATGGGGAGCGCATCCAAAGACTCGGTAGAGTTGGGCGACACAAGGAAGGAGTAGCACTT
CGAATTGGCCAAACAAATAAAACACTGGTTGAAATTCCAGAAATGGTTGCCACTGAAGCTGCCTTTCTATGCTTC
ATGTACAATTTGCCAGTGACAACACAGAGTGTTTCAACCACACTGCTGGAAAATGCCACATTATTACAAGCTAGA
ACTATGGCACAGTTTGAGCTATCATATTTTTACACAATTAATTTTGTGCGATTTGATGGTAGTATGCATCCAGTC
ATACATGACAAGCTGAAGCGCTTTAAGCTACACACTTGTGAGACATTCCTCAATAAGTTGGCGATCCCAAATAAA
GGCTTATCCTCTTGGCTTACGAGTGGAGAGTATAAGCGACTTGGTTACATAGCAGAGGATGCTGGCATAAGAATC
CCATTCGTGTGCAAAGAAATTCCAGACTCCTTGCATGAGGAAATTTGGCACATTGTAGTCGCCCATAAAGGTGAC
TCGGGTATTGGGAGGCTCACTAGCGTACAGGCAGCAAAGGTTGTTTATACTCTGCAAACGGATGTGCACTCAATT
GCGAGGACTCTAGCATGCATCAATAGACTCATAGCACATGAACAAATGAAGCAGAGTCATTTTGAAGCCGCAACT
GGGAGAGCATTTTCCTTCACAAATTACTCAATACAAAGCATATTTGACACGCTGAAAGCAAATTATGCTACAAAG
CATACGAAAGAAAATATTGCAGTGCTTCAGCAGGCAAAAGATCAATTGCTAGAGTTTTCGAACCTAGCAAAGGAT
CAAGATGTCACGGGTATCATCCAAGACTTCAATCACCTGGAAACTATCTATCTCCAATCAGATAGCGAAGTGGCT
AAGCATCTGAAGCTTAAAAGTCACTGGAATAAAAGCCAAATCACTAGGGACATCATAATAGCTTTGTCTGTGTTA
ATTGGTGGTGGATGGATGCTTGCAACGTACTTCAAGGACAAGTTCAATGAACCAGTCTATTTCCAAGGGAAGAAG
AATCAGAAGCACAAGCTTAAGATGAGAGAGGCGCGTGGGGCTAGAGGGCAATATGAGGTTGCAGCGGAGCCAGAG
GCGCTAGAACATTACTTTGGAAGCGCATATAATAACAAAGGAAAGCGCAAGGGCACCACGAGAGGAATGGGTGCA
AAGTCTCGGAAATTCATAAACATGTATGGGTTTGATCCAACTGATTTTTCATACATTAGGTTTGTGGATCCATTG
ACAGGTCACACTATTGATGAGTCCACAAACGCACCTATTGATTTAGTGCAGCATGAGTTTGGAAAGGTTAGAACA
CGCATGTTAATTGACGATGAGATAGAGCCTCAAAGTCTTAGCACCCACACCACAATCCATGCTTATTTGGTGAAT
AGTGGCACGAAGAAAGTTCTTAAGGTTGATTTAACACCACACTCGTCGCTACGTGCGAGTGAGAAATCAACAGCA
ATAATGGGATTTCCTGAAAGGGAGAATGAATTGCGTCAAACCGGCATGGCAGTGCCAGTGGCTTATGATCAATTG
CCACCAAAGAGTGAGGACTTGACGTTTGAAGGAGAAAGCTTGTTTAAGGGACCACGTGATTACAACCCGATATCG
AGCACCATTTGTCACTTGACGAATGAATCTGATGGGCACACAACATCGTTGTATGGTATTGGATTTGGTCCCTTC
ATCATTACAAACAAGCACTTGTTTAGAAGAAATAATGGAACACTGTTGGTCCAATCACTACATGGTGTATTCAAG
GTCAAGAACACCACGACTTTGCAACAACACCTCATTGATGGGAGGGACATGATAATTATTCGCATGCCTAAGGAT
TTCCCACCATTTCCTCAAAAGCTGAAATTTAGAGAGCCACAAAGGGAAGAGCGCATATGTCTTGTGACAACCAAC
TTCCAAACTAAGAGCATGTCTAGCATGGTGTCAGACACTAGTTGCACATTCCCTTCATCTGATGGCATATTCTGG
AAGCATTGGATTCAAACCAAGGATGGGCAGTGTGGCAGTCCATTAGTATCAACTAGAGATGGGTTCATTGTTGGT
ATACACTCAGCATCGAATTTCACCAACACAAACAATTATTTCACAAGCGTGCCGAAAAACTTCATGGAATTGTTG
ACAAATCAGGAGGCGCAGCAGTGGGTTAGTGGTTGGCGATTAAATGCTGACTCAGTATTGTGGGGGGGCCATAAA
GTTTTCATGAGCAAACCTGAAGAGCCTTTTCAGCCAGTTAAGGAAGCGACTCAACTCATGAGTGAATTGGTGTAC
TCGCAAAGTGGCACTGTGGGTGCTGGTGTTGACGCTGGTAAGAAGAAAGATCAAAAGGATGATAAAGTCGCTGAG
CAGGCTTCAAAGGATAGGGATGTTAATGCTGGAACTTCAGGAACATTCTCAGTTCCACGAATAAATGCTATGGCC
ACAAAACTTCAATATCCAAGGATGAGGGGAGAGGTGGTTGTAAACTTGAATCACCTTTTAGGATACAAGCCACAG
CAAATTGATTTGTCAAATGCTCGAGCCACACATGAGCAGTTTGCCGCGTGGCATCAGGCAGTGATGACAGCCTAT
GGAGTGAATGAAGAGCAAATGAAAATATTGCTAAATGGATTTATGGTGTGGTGCATAGAAAATGGGACTTCCCCA
AATTTGAACGGAACTTGGGTTATGATGGATGGTGAGGAGCAAGTTTCATACCCGCTGAAACCAATGGTTGAAAAC
GCGCAGCCAACACTGAGGCAAATTATGACACACTTCAGTGACCTGGCTGAAGCGTATATTGAGATGAGGAATAGG
GAGCGACCATACATGCCTAGGTATGGTCTACAGAGAAACATTACAGACATGAGTTTGTCACGCTATGCGTTCGAC
TTCTATGAGCTAACTTCAAAAACACCTGTTAGAGCGAGGGAGGCGCATATGCAAATGAAAGCTGCTGCAGTACGA
AACAGTGGAACTAGGTTATTTGGTCTTGATGGCAACGTGGGTACTGCAGAGGAAGACACTGAACGGCACACAGCG
CACGATGTGAACCGTAACATGCACACACTATTAGGGGTCCGCCAGTGATAGTTTCTGCGTGTCTTTGCTTTCCGC
TTTTAAGCTTATTGTAATATATATGAATAGCTATTCACAGTGGGACTTGGTCTTGTGTTGAATGGTATCTTATAT
GTTTTAATATGTCTTATTAGTCTCATTACTTAGGCGAACGACAAAGTGAGGTCACCTCGGTCTAATTCTCCTATG
TAGTGCGAGAAAAAAAAAAAAAAAAAAAAAAAAAAAAAAAAAAAAAAAAAA

>TEVΔNIb-BIE (insert between possitions 6981-6982 of TEVΔNIb)

GGGGAGAAGATGAATAATCCGTCGTTACTCAATCATGCGGTCGAAACGATGGCAGTTGGCTCGAAAAGTTTTGCG
ACAGCCTCAAAGTTATTTGATGCAAAAACCCGGCGCAGCGTACTGATGCTCTACGCCTGGTGCCGCCATTGTGAC
GATGTTATTGACGATCAGACGCTGGGCTTTCAGGCCCGGCAGCCTGCCTTACAAACGCCCGAACAACGTCTGATG
CAACTTGAGATGAAAACGCGCCAGGCCTATGCAGGATCGCAGATGCACGAACCGGCGTTTGCGGCTTTTCAGGAA
GTGGCTATGGCTCATGATATCGCCCCGGCTTACGCGTTTGATCATCTGGAAGGCTTCGCCATGGATGTACGCGAA
GCGCAATACAGCCAACTGGATGATACGCTGCGCTATTGCTATCACGTTGCAGGCGTTGTCGGCTTGATGATGGCG
CAAATCATGGGCGTGCGGGATAACGCCACGCTGGACCGCGCCTGTGACCTTGGGCTGGCATTTCAGTTGACCAAT
ATTGCTCGCGATATTGTGGACGATGCGCATGCGGGCCGCTGTTATCTGCCGGCAAGCTGGCTGGAGCATGAAGGT
CTGAACAAAGAGAATTATGCGGCACCTGAAAACCGTCAGGCGCTGAGCCGTATCGCCCGTCGTTTGGTGCAGGAA
GCAGAACCTTACTATTTGTCTGCCACAGCCGGCCTGGCAGGGTTGCCCCTGCGTTCCGCCTGGGCAATCGCTACG
GCGAAGCAGGTTTACCGGAAAATAGGTGTCAAAGTTGAACAGGCCGGTCAGCAAGCCTGGGATCAGCGGCAGTCA
ACGACCACGCCCGAAAAATTAACGCTGCTGCTGGCCGCCTCTGGTCAGGCCCTTACTTCCCGGATGCGGGCTCAT
CCTCCCCGCCCTGCGCATCTCTGGCAGCGCCCGCTCACGACTGAAAACTTGTACTTCCAAAGCGGCACTATGAAA
CCAACTACGGTAATTGGTGCAGGCTTCGGTGGCCTGGCACTGGCAATTCGTCTACAAGCTGCGGGGATCCCCGTC
TTACTGCTTGAACAACGTGATAAACCCGGCGGTCGGGCTTATGTCTACGAGGATCAGGGGTTTACCTTTGATGCA
GGCCCGACGGTTATCACCGATCCCAGTGCCATTGAAGAACTGTTTGCACTGGCAGGAAAACAGTTAAAAGAGTAT
GTCGAACTGCTGCCGGTTACGCCGTTTTACCGCCTGTGTTGGGAGTCAGGGAAGGTCTTTAATTACGATAACGAT
CAAACCCGGCTCGAAGCGCAGATTCAGCAGTTTAATCCCCGCGATGTCGAAGGTTATCGTCAGTTTCTGGACTAT
TCACGCGCGGTGTTTAAAGAAGGCTATCTAAAGCTCGGTACTGTCCCTTTTTTATCGTTCAGAGACATGCTTCGC
GCCGCACCTCAACTGGCGAAACTGCAGGCATGGAGAAGCGTTTACAGTAAGGTTGCCAGTTACATCGAAGATGAA
CATCTGCGCCAGGCGTTTTCTTTCCACTCGCTGTTGGTGGGCGGCAATCCCTTCGCCACCTCATCCATTTATACG
TTGATACACGCGCTGGAGCGTGAGTGGGGCGTCTGGTTTCCGCGTGGCGGCACCGGCGCATTAGTTCAGGGGATG
ATAAAGCTGTTTCAGGATCTGGGTGGCGAAGTCGTGTTAAACGCCAGAGTCAGCCATATGGAAACGACAGGAAAC
AAGATTGAAGCCGTGCATTTAGAGGACGGTCGCAGGTTCCTGACGCAAGCCGTCGCGTCAAATGCAGATGTGGTT
CATACCTATCGCGACCTGTTAAGCCAGCACCCTGCCGCGGTTAAGCAGTCCAACAAACTGCAGACTAAGCGCATG
AGTAACTCTCTGTTTGTGCTCTATTTTGGTTTGAATCACCATCATGATCAGCTCGCGCATCACACGGTTTGTTTC
GGCCCGCGTTACCGCGAGCTGATTGACGAAATTTTTAATCATGATGGCCTCGCAGAGGACTTCTCACTTTATCTG
CACGCGCCCTGTGTCACGGATTCGTCACTGGCGCCTGAAGGTTGCGGCAGTTACTATGTGTTGGCGCCGGTGCCG
CATTTAGGCACCGCGAACCTCGACTGGACGGTTGAGGGGCCAAAACTACGCGACCGTATTTTTGCGTACCTTGAG
CAGCATTACATGCCTGGCTTACGGAGTCAGCTGGTCACGCACCGGATGTTTACGCCGTTTGATTTTCGCGACCAG
CTTAATGCCTATCATGGCTCAGCCTTTTCTGTGGAGCCCGTTCTTACCCAGAGCGCCTGGTTTCGGCCGCATAAC
CGCGATAAAACCATTACTAATCTCTACCTGGTCGGCGCAGGCACGCATCCCGGCGCAGGCATTCCTGGCGTCATC
GGCTCGGCAAAAGCGACAGCAGGTTTGATGCTGGAGGATCTGATAACTACAGAGAACCTCTACTTTCAATCAGGT
ACAATGACGGTCTGCGCAAAAAAACACGTTCATCTCACTCGCGATGCTGCGGAGCAGTTACTGGCTGATATTGAT
CGACGCCTTGATCAGTTATTGCCCGTGGAGGGAGAACGGGATGTTGTGGGTGCCGCGATGCGTGAAGGTGCGCTG
GCACCGGGAAAACGTATTCGCCCCATGTTGCTGTTGCTGACCGCCCGCGATCTGGGTTGCGCTGTCAGCCATGAC
GGATTACTGGATTTGGCCTGTGCGGTGGAAATGGTCCACGCGGCTTCGCTGATCCTTGACGATATGCCCTGCATG
GACGATGCGAAGCTGCGGCGCGGACGCCCTACCATTCATTCTCATTACGGAGAGCATGTGGCAATACTGGCGGCG
GTTGCCTTGCTGAGTAAAGCCTTTGGCGTAATTGCCGATGCAGATGGCCTCACGCCGCTGGCAAAAAATCGGGCG
GTTTCTGAACTGTCAAACGCCATCGGCATGCAAGGATTGGTTCAGGGTCAGTTCAAGGATCTGTCTGAAGGGGAT
AAGCCGCGCAGCGCTGAAGCTATTTTGATGACGAATCACTTTAAAACCAGCACGCTGTTTTGTGCCTCCATGCAG
ATGGCCTCGATTGTTGCGAATGCCTCCAGCGAAGCGCGTGATTGCCTGCATCGTTTTTCACTTGATCTTGGTCAG
GCATTTCAACTGCTGGACGATTTGACCGATGGCATGACCGACACCGGTAAGGATAGCAATCAGGACGCCGGTAAA
TCGACGCTGGTCAATCTGTTAGGCCCGAGGGCGGTTGAAGAACGTCTGAGACAACATCTTCAGCTTGCCAGTGAG
CATCTCTCTGCGGCCTGCCAACACGGGCACGCCACTCAACATTTTATTCAGGCCTGGTTTGACAAAAAACTCGCT
GCCGTCAGTACGACTGAGAATCTTTATTTTCAG

>TEVΔNIb-E (insert between possitions 6981-6982 of TEVΔNIb)

GGGGAGAAGATGACGGTCTGCGCAAAAAAACACGTTCATCTCACTCGCGATGCTGCGGAGCAGTTACTGGCTGAT
ATTGATCGACGCCTTGATCAGTTATTGCCCGTGGAGGGAGAACGGGATGTTGTGGGTGCCGCGATGCGTGAAGGT
GCGCTGGCACCGGGAAAACGTATTCGCCCCATGTTGCTGTTGCTGACCGCCCGCGATCTGGGTTGCGCTGTCAGC
CATGACGGATTACTGGATTTGGCCTGTGCGGTGGAAATGGTCCACGCGGCTTCGCTGATCCTTGACGATATGCCC
TGCATGGACGATGCGAAGCTGCGGCGCGGACGCCCTACCATTCATTCTCATTACGGAGAGCATGTGGCAATACTG
GCGGCGGTTGCCTTGCTGAGTAAAGCCTTTGGCGTAATTGCCGATGCAGATGGCCTCACGCCGCTGGCAAAAAAT
CGGGCGGTTTCTGAACTGTCAAACGCCATCGGCATGCAAGGATTGGTTCAGGGTCAGTTCAAGGATCTGTCTGAA
GGGGATAAGCCGCGCAGCGCTGAAGCTATTTTGATGACGAATCACTTTAAAACCAGCACGCTGTTTTGTGCCTCC
ATGCAGATGGCCTCGATTGTTGCGAATGCCTCCAGCGAAGCGCGTGATTGCCTGCATCGTTTTTCACTTGATCTT
GGTCAGGCATTTCAACTGCTGGACGATTTGACCGATGGCATGACCGACACCGGTAAGGATAGCAATCAGGACGCC
GGTAAATCGACGCTGGTCAATCTGTTAGGCCCGAGGGCGGTTGAAGAACGTCTGAGACAACATCTTCAGCTTGCC

AGTGAGCATCTCTCTGCGGCCTGCCAACACGGGCACGCCACTCAACATTTTATTCAGGCCTGGTTTGACAAAAAA
CTCGCTGCCGTCAGTACGACTGAGAATCTTTATTTTCAG

>TEVΔNIb-B (insert between possitions 6981-6982 of TEVΔNIb)
GGGGAGAAGATGAATAATCCGTCGTTACTCAATCATGCGGTCGAAACGATGGCAGTTGGCTCGAAAAGTTTTGCG
ACAGCCTCAAAGTTATTTGATGCAAAAACCCGGCGCAGCGTACTGATGCTCTACGCCTGGTGCCGCCATTGTGAC
GATGTTATTGACGATCAGACGCTGGGCTTTCAGGCCCGGCAGCCTGCCTTACAAACGCCCGAACAACGTCTGATG
CAACTTGAGATGAAAACGCGCCAGGCCTATGCAGGATCGCAGATGCACGAACCGGCGTTTGCGGCTTTTCAGGAA
GTGGCTATGGCTCATGATATCGCCCCGGCTTACGCGTTTGATCATCTGGAAGGCTTCGCCATGGATGTACGCGAA
GCGCAATACAGCCAACTGGATGATACGCTGCGCTATTGCTATCACGTTGCAGGCGTTGTCGGCTTGATGATGGCG
CAAATCATGGGCGTGCGGGATAACGCCACGCTGGACCGCGCCTGTGACCTTGGGCTGGCATTTCAGTTGACCAAT
ATTGCTCGCGATATTGTGGACGATGCGCATGCGGGCCGCTGTTATCTGCCGGCAAGCTGGCTGGAGCATGAAGGT
CTGAACAAAGAGAATTATGCGGCACCTGAAAACCGTCAGGCGCTGAGCCGTATCGCCCGTCGTTTGGTGCAGGAA
GCAGAACCTTACTATTTGTCTGCCACAGCCGGCCTGGCAGGGTTGCCCCTGCGTTCCGCCTGGGCAATCGCTACG
GCGAAGCAGGTTTACCGGAAAATAGGTGTCAAAGTTGAACAGGCCGGTCAGCAAGCCTGGGATCAGCGGCAGTCA
ACGACCACGCCCGAAAAATTAACGCTGCTGCTGGCCGCCTCTGGTCAGGCCCTTACTTCCCGGATGCGGGCTCAT
CCTCCCCGCCCTGCGCATCTCTGGCAGCGCCCGCTCACGACTGAGAATCTTTATTTTCAG

>TEVΔNIb-I (insert between possitions 6981-6982 of TEVΔNIb)
GGGGAGAAGATGAAACCAACTACGGTAATTGGTGCAGGCTTCGGTGGCCTGGCACTGGCAATTCGTCTACAAGCT
GCGGGGATCCCCGTCTTACTGCTTGAACAACGTGATAAACCCGGCGGTCGGGCTTATGTCTACGAGGATCAGGGG
TTTACCTTTGATGCAGGCCCGACGGTTATCACCGATCCCAGTGCCATTGAAGAACTGTTTGCACTGGCAGGAAAA
CAGTTAAAAGAGTATGTCGAACTGCTGCCGGTTACGCCGTTTTACCGCCTGTGTTGGGAGTCAGGGAAGGTCTTT
AATTACGATAACGATCAAACCCGGCTCGAAGCGCAGATTCAGCAGTTTAATCCCCGCGATGTCGAAGGTTATCGT
CAGTTTCTGGACTATTCACGCGCGGTGTTTAAAGAAGGCTATCTAAAGCTCGGTACTGTCCCTTTTTTATCGTTC
AGAGACATGCTTCGCGCCGCACCTCAACTGGCGAAACTGCAGGCATGGAGAAGCGTTTACAGTAAGGTTGCCAGT
TACATCGAAGATGAACATCTGCGCCAGGCGTTTTCTTTCCACTCGCTGTTGGTGGGCGGCAATCCCTTCGCCACC
TCATCCATTTATACGTTGATACACGCGCTGGAGCGTGAGTGGGGCGTCTGGTTTCCGCGTGGCGGCACCGGCGCA
TTAGTTCAGGGGATGATAAAGCTGTTTCAGGATCTGGGTGGCGAAGTCGTGTTAAACGCCAGAGTCAGCCATATG
GAAACGACAGGAAACAAGATTGAAGCCGTGCATTTAGAGGACGGTCGCAGGTTCCTGACGCAAGCCGTCGCGTCA
AATGCAGATGTGGTTCATACCTATCGCGACCTGTTAAGCCAGCACCCTGCCGCGGTTAAGCAGTCCAACAAACTG
CAGACTAAGCGCATGAGTAACTCTCTGTTTGTGCTCTATTTTGGTTTGAATCACCATCATGATCAGCTCGCGCAT
CACACGGTTTGTTTCGGCCCGCGTTACCGCGAGCTGATTGACGAAATTTTTAATCATGATGGCCTCGCAGAGGAC
TTCTCACTTTATCTGCACGCGCCCTGTGTCACGGATTCGTCACTGGCGCCTGAAGGTTGCGGCAGTTACTATGTG
TTGGCGCCGGTGCCGCATTTAGGCACCGCGAACCTCGACTGGACGGTTGAGGGGCCAAAACTACGCGACCGTATT
TTTGCGTACCTTGAGCAGCATTACATGCCTGGCTTACGGAGTCAGCTGGTCACGCACCGGATGTTTACGCCGTTT
GATTTTCGCGACCAGCTTAATGCCTATCATGGCTCAGCCTTTTCTGTGGAGCCCGTTCTTACCCAGAGCGCCTGG
TTTCGGCCGCATAACCGCGATAAAACCATTACTAATCTCTACCTGGTCGGCGCAGGCACGCATCCCGGCGCAGGC
ATTCCTGGCGTCATCGGCTCGGCAAAAGCGACAGCAGGTTTGATGCTGGAGGATCTGATAACGACTGAGAATCTT
TATTTTCAG

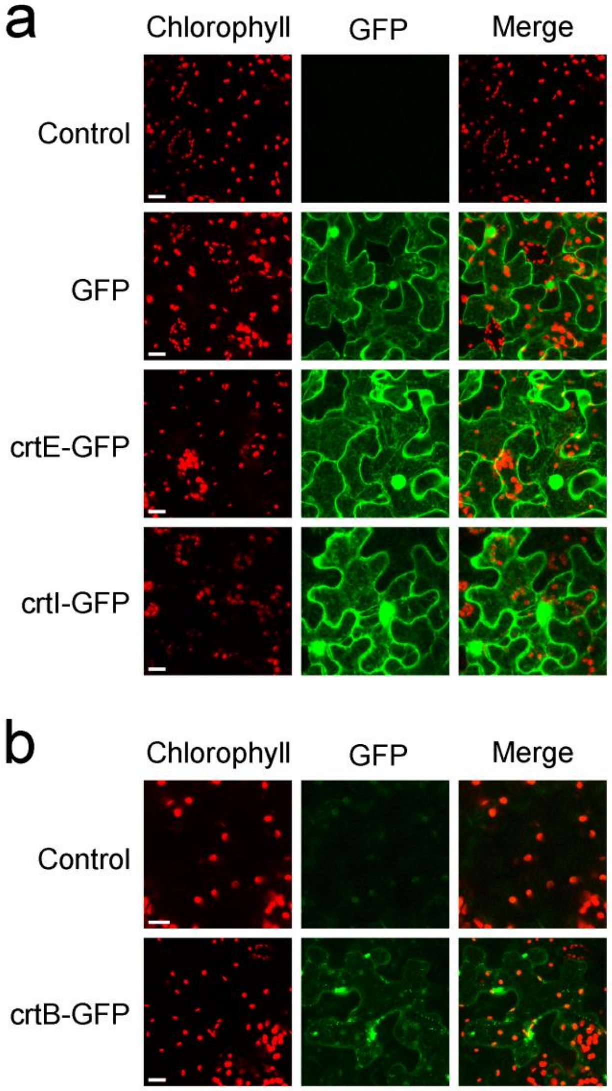

Figure S2. Subcellular localization of the indicated GFP-tagged proteins in agroinfiltrated *N. benthamiana* leaves visualized by confocal microscopy. Mock-treated controls and an empty vector control (with only the GFP protein) are also shown. Images were acquired using a laser intensity of 25% and a Smart Gain of 426 (**a**) or a laser intensity of 27% and a Smart Gain of 1069 (**b**). Chlorophyll autofluorescence (in red); GFP fluorescence (in green) and merged images of both are shown. Bar, 10 μm.

Figure S3. Full sequences of virus clones TEV-wt, TEV-crtB, TEV-Ros1, TMV-crtB, PVX-crtB, ZYMV-wt, ZYMV-crtB and ZYMV-Ros1. Some details of each particular virus clone are indicated below each sequence.

>TEV-wt

AAAATAACAAATCTCAACACAACATATACAAAACAAACGAATCTCAAGCAATCAAGCATTCTACTTCTATTGCAG
CAATTTAAATCATTTCTTTTAAAGCAAAAGCAATTTTCTGAAAATTTTCACCATTTACGAACGATAGCCATGGCA
CTCATCTTTGGCACAGTCAACGCTAACATCCTGAAGGAAGTGTTCGGTGGAGCTCGTATGGCTTGCGTTACCAGC
GCACATATGGCTGGAGCGAATGGAAGCATTTTGAAGAAGGCAGAAGAAACCTCTCGTGCAATCATGCACAAACCA
GTGATCTTCGGAGAAGACTACATTACCGAGGCAGACTTGCCTTACACACCACTCCATTTAGAGGTCGATGCTGAA
ATGGAGCGGATGTATTATCTTGGTCGTCGCGCGCTCACCCATGGCAAGAGACGCAAAGTTTCTGTGAATAACAAG
AGGAACAGGAGAAGGAAAGTGGCCAAAACGTACGTGGGGCGTGATTCCATTGTTGAGAAGATTGTAGTGCCCCAC
ACCGAGAGAAAGGTTGATACCACAGCAGCAGTGGAAGACATTTGCAATGAAGCTACCACTCAACTTGTGCATAAT
AGTATGCCAAAGCGTAAGAAGCAGAAAAACTTCTTGCCCGCCACTTCACTAAGTAACGTGTATGCCCAAACTTGG
AGCATAGTGCGCAAACGCCATATGCAGGTGGAGATCATTAGCAAGAAGAGCGTCCGAGCGAGGGTCAAGAGATTT
GAGGGCTCGGTGCAATTGTTCGCAAGTGTGCGTCACATGTATGGCGAGAGGAAAAGGGTGGACTTACGTATTGAC
AACTGGCAGCAAGAGACACTTCTAGACCTTGCTAAAAGATTTAAGAATGAGAGAGTGGATCAATCGAAGCTCACT
TTTGGTTCAAGTGGCCTAGTTTTGAGGCAAGGCTCGTACGGACCTGCGCATTGGTATCGACATGGTATGTTCATT
GTACGCGGTCGGTCGGATGGGATGTTGGTGGATGCTCGTGCGAAGGTAACGTTCGCTGTTTGTCACTCAATGACA
CATTATAGCGACAAATCAATCTCTGAGGCATTCTTCATACCATACTCTAAGAAATTCTTGGAGTTGAGGCCAGAT
GGAATCTCCCATGAGTGTACAAGAGGAGTATCAGTTGAGCGGTGCGGTGAGGTGGCTGCAATCCTGACACAAGCA
CTTTCACCGTGTGGTAAGATCACATGCAAACGTTGCATGGTTGAAACACCTGACATTGTTGAGGGTGAGTCGGGA
GACAGTGTCACCAACCAAGGTAAGCTCCTAGCAATGCTGAAAGAACAGTATCCAGATTTCCCAATGGCCGAGAAA
CTACTCACAAGGTTTTTGCAACAGAAATCACTAGTAAATACAAATTTGACAGCCTGCGTGAGCGTCAAACAACTC
ATTGGTGACCGCAAACAAGCTCCATTCACACACGTACTGGCTGTCAGCGAAATTCTGTTTAAAGGCAATAAACTA
ACAGGGGCCGATCTCGAAGAGGCAAGCACACATATGCTTGAAATAGCAAGGTTCTTGAACAATCGCACTGAAAAT
ATGCGCATTGGCCACCTTGGTTCTTTCAGAAATAAAATCTCATCGAAGGCCCATGTGAATAACGCACTCATGTGT
GATAATCAACTTGATCAGAATGGGAATTTTATTTGGGGACTAAGGGGTGCACACGCAAAGAGGTTTCTTAAAGGA
TTTTTCACTGAGATTGACCCAAATGAAGGATACGATAAGTATGTTATCAGGAAACATATCAGGGGTAGCAGAAAG
CTAGCAATTGGCAATTTGATAATGTCAACTGACTTCCAGACGCTCAGGCAACAAATTCAAGGCGAAACTATTGAG
CGTAAAGAAATTGGGAATCACTGCATTTCAATGCGGAATGGTAATTACGTGTACCCATGTTGTTGTGTTACTCTT
GAAGATGGTAAGGCTCAATATTCGGATCTAAAGCATCCAACGAAGAGACATCTGGTCATTGGCAACTCTGGCGAT
TCAAAGTACCTAGACCTTCCAGTTCTCAATGAAGAGAAAATGTATATAGCTAATGAAGGTTATTGCTACATGAAC
ATTTTCTTTGCTCTACTAGTGAATGTCAAGGAAGAGGATGCAAAGGACTTCACCAAGTTTATAAGGGACACAATT
GTTCCAAAGCTTGGAGCGTGGCCAACAATGCAAGATGTTGCAACTGCATGCTACTTACTTTCCATTCTTTACCCA
GATGTCCTGAGTGCTGAATTACCCAGAATTTTGGTTGATCATGACAACAAAACAATGCATGTTTTGGATTCGTAT
GGGTCTAGAACGACAGGATACCACATGTTGAAAATGAACACAACATCCCAGCTAATTGAATTCGTTCATTCAGGT
TTGGAATCCGAAATGAAAACTTACAATGTTGGAGGGATGAACCGAGATATGGTCACACAAGGTGCAATTGAGATG
TTGATCAAGTCCATATACAAACCACATCTCATGAAGCAGTTACTTGAGGAGGAGCCATACATAATTGTCCTGGCA
ATAGTCTCCCCTTCAATTTTAATTGCCATGTACAACTCTGGAACTTTTGAGCAGGCGTTACAAATGTGGTTGCCA
AATACAATGAGGTTAGCTAACCTCGCTGCCATCTTGTCAGCCTTGGCGCAAAAGTTAACTTTGGCAGACTTGTTC
GTCCAGCAGCGTAATTTGATTAATGAGTATGCGCAGGTAATTTTGGACAATCTGATTGACGGTGTCAGGGTTAAC
CATTCGCTATCCCTAGCAATGGAAATTGTTACTATTAAGCTGGCCACCCAAGAGATGGACATGGCGTTGAGGGAA
GGTGGCTATGCTGTGACCTCTGAAAAGGTGCATGAAATGTTGGAAAAAAACTATGTAAAGGCTTTGAAGGATGCA
TGGGACGAATTAACTTGGTTGGAAAAATTCTCCGCAATCAGGCATTCAAGAAAGCTCTTGAAATTTGGGCGAAAG
CCTTTAATCATGAAAAACACCGTAGATTGCGGCGGACATATAGACTTGTCTGTGAAATCGCTTTTCAAGTTCCAC
TTGGAACTCCTGAAGGGAACCATCTCAAGAGCCGTAAATGGTGGTGCAAGAAAGGTAAGAGTAGCGAAGAATGCC
ATGACAAAAGGGGTTTTTCTCAAAATCTACAGCATGCTTCCTGACGTCTACAAGTTTATCACAGTCTCGAGTGTC
CTTTCCTTGTTGTTGACATTCTTATTTCAAATTGACTGCATGATAAGGGCACACCGAGAGGCGAAGGTTGCTGCA
CAGTTGCAGAAAGAGAGCGAGTGGGACAATATCATCAATAGAACTTTCCAGTATTCTAAGCTTGAAAATCCTATT
GGCTATCGCTCTACAGCGGAGGAAAGACTCCAATCAGAACACCCCGAGGCTTTCGAGTACTACAAGTTTTGCATT
GGAAAGGAAGACCTCGTTGAACAGGCAAAACAACCGGAGATAGCATACTTTGAAAAGATTATAGCTTTCATCACA
CTTGTATTAATGGCTTTTGACGCTGAGCGGAGTGATGGAGTGTTCAAGATACTCAATAAGTTCAAAGGAATACTG
AGCTCAACGGAGAGGGAGATCATCTACACGCAGAGTTTGGATGATTACGTTACAACCTTTGATGACAATATGACA
ATCAACCTCGAGTTGAATATGGATGAACTCCACAAGACGAGCCTTCCTGGAGTCACTTTTAAGCAATGGTGGAAC
AACCAAATCAGCCGAGGCAACGTGAAGCCACATTATAGAACTGAGGGGCACTTCATGGAGTTTACCAGAGATACT
GCGGCATCGGTTGCCAGCGAGATATCACACTCACCCGCAAGAGATTTTCTTGTGAGAGGTGCTGTTGGATCTGGA
AAATCCACAGGACTTCCATACCATTTATCAAAGAGAGGGAGAGTGTTAATGCTTGAGCCTACCAGACCACTCACA

GATAACGTGCACAAGCAACTGAGAAGTGAACCATTTAACTGCTTCCCAACTTTGAGGATGAGAGGGAAGTCAACT
TTTGGGTCATCACCGATTACAGTCATGACTAGTGGATTCGCTTTACACCATTTTGCACGAAACATAGCTGAGGTA
AAAACATACGATTTTGTCATAATTGATGAATGTCATGTGAATGATGCTTCTGCTATAGCGTTTAGGAATCTACTG
TTTGAACATGAATTTGAAGGAAAAGTCCTCAAAGTGTCAGCCACACCACCAGGTAGAGAAGTTGAATTCACAACT
CAGTTTCCCGTGAAACTCAAGATAGAAGAGGCTCTTAGCTTTCAGGAATTTGTAAGTTTACAAGGGACAGGTGCC
AACGCCGATGTGATTAGTTGTGGCGACAACATACTAGTATATGTTGCTAGCTACAATGATGTTGATAGTCTTGGC
AAGCTCCTTGTGCAAAAGGGATACAAAGTGTCGAAGATTGATGGAAGAACAATGAAGAGTGGAGGAACTGAAATA
ATCACTGAAGGTACTTCAGTGAAAAAGCATTTCATAGTCGCAACTAATATTATTGAGAATGGTGTAACCATTGAC
ATTGATGTAGTTGTGGATTTTGGGACTAAGGTTGTACCAGTTTTGGATGTGGACAATAGAGCGGTGCAGTACAAC
AAAACTGTGGTGAGTTATGGGGAGCGCATCCAAAGACTCGGTAGAGTTGGGCGACACAAGGAAGGAGTAGCACTT
CGAATTGGCCAAACAAATAAAACACTGGTTGAAATTCCAGAAATGGTTGCCACTGAAGCTGCCTTTCTATGCTTC
ATGTACAATTTGCCAGTGACAACACAGAGTGTTTCAACCACACTGCTGGAAAATGCCACATTATTACAAGCTAGA
ACTATGGCACAGTTTGAGCTATCATATTTTTACACAATTAATTTTGTGCGATTTGATGGTAGTATGCATCCAGTC
ATACATGACAAGCTGAAGCGCTTTAAGCTACACACTTGTGAGACATTCCTCAATAAGTTGGCGATCCCAAATAAA
GGCTTATCCTCTTGGCTTACGAGTGGAGAGTATAAGCGACTTGGTTACATAGCAGAGGATGCTGGCATAAGAATC
CCATTCGTGTGCAAAGAAATTCCAGACTCCTTGCATGAGGAAATTTGGCACATTGTAGTCGCCCATAAAGGTGAC
TCGGGTATTGGGAGGCTCACTAGCGTACAGGCAGCAAAGGTTGTTTATACTCTGCAAACGGATGTGCACTCAATT
GCGAGGACTCTAGCATGCATCAATAGACTCATAGCACATGAACAAATGAAGCAGAGTCATTTTGAAGCCGCAACT
GGGAGAGCATTTTCCTTCACAAATTACTCAATACAAAGCATATTTGACACGCTGAAAGCAAATTATGCTACAAAG
CATACGAAAGAAAATATTGCAGTGCTTCAGCAGGCAAAAGATCAATTGCTAGAGTTTTCGAACCTAGCAAAGGAT
CAAGATGTCACGGGTATCATCCAAGACTTCAATCACCTGGAAACTATCTATCTCCAATCAGATAGCGAAGTGGCT
AAGCATCTGAAGCTTAAAAGTCACTGGAATAAAAGCCAAATCACTAGGGACATCATAATAGCTTTGTCTGTGTTA
ATTGGTGGTGGATGGATGCTTGCAACGTACTTCAAGGACAAGTTCAATGAACCAGTCTATTTCCAAGGGAAGAAG
AATCAGAAGCACAAGCTTAAGATGAGAGAGGCGCGTGGGGCTAGAGGGCAATATGAGGTTGCAGCGGAGCCAGAG
GCGCTAGAACATTACTTTGGAAGCGCATATAATAACAAAGGAAAGCGCAAGGGCACCACGAGAGGAATGGGTGCA
AAGTCTCGGAAATTCATAAACATGTATGGGTTTGATCCAACTGATTTTTCATACATTAGGTTTGTGGATCCATTG
ACAGGTCACACTATTGATGAGTCCACAAACGCACCTATTGATTTAGTGCAGCATGAGTTTGGAAAGGTTAGAACA
CGCATGTTAATTGACGATGAGATAGAGCCTCAAAGTCTTAGCACCCACACCACAATCCATGCTTATTTGGTGAAT
AGTGGCACGAAGAAAGTTCTTAAGGTTGATTTAACACCACACTCGTCGCTACGTGCGAGTGAGAAATCAACAGCA
ATAATGGGATTTCCTGAAAGGGAGAATGAATTGCGTCAAACCGGCATGGCAGTGCCAGTGGCTTATGATCAATTG
CCACCAAAGAGTGAGGACTTGACGTTTGAAGGAGAAAGCTTGTTTAAGGGACCACGTGATTACAACCCGATATCG
AGCACCATTTGTCACTTGACGAATGAATCTGATGGGCACACAACATCGTTGTATGGTATTGGATTTGGTCCCTTC
ATCATTACAAACAAGCACTTGTTTAGAAGAAATAATGGAACACTGTTGGTCCAATCACTACATGGTGTATTCAAG
GTCAAGAACACCACGACTTTGCAACAACACCTCATTGATGGGAGGGACATGATAATTATTCGCATGCCTAAGGAT
TTCCCACCATTTCCTCAAAAGCTGAAATTTAGAGAGCCACAAAGGGAAGAGCGCATATGTCTTGTGACAACCAAC
TTCCAAACTAAGAGCATGTCTAGCATGGTGTCAGACACTAGTTGCACATTCCCTTCATCTGATGGCATATTCTGG
AAGCATTGGATTCAAACCAAGGATGGGCAGTGTGGCAGTCCATTAGTATCAACTAGAGATGGGTTCATTGTTGGT
ATACACTCAGCATCGAATTTCACCAACACAAACAATTATTTCACAAGCGTGCCGAAAAACTTCATGGAATTGTTG
ACAAATCAGGAGGCGCAGCAGTGGGTTAGTGGTTGGCGATTAAATGCTGACTCAGTATTGTGGGGGGGCCATAAA
GTTTTCATGAGCAAACCTGAAGAGCCTTTTCAGCCAGTTAAGGAAGCGACTCAACTCATGAGTGAATTGGTGTAC
TCGCAAGGGGAGAAGAGGAAATGGGTCGTGGAAGCACTGTCAGGGAACTTGAGGCCAGTGGCTGAGTGTCCCAGT
CAGTTAGTCACAAAGCATGTGGTTAAAGGAAAGTGTCCCCTCTTTGAGCTCTACTTGCAGTTGAATCCAGAAAAG
GAAGCATATTTTAAACCGATGATGGGAGCATATAAGCCAAGTCGACTTAATAGAGAGGCGTTCCTCAAGGACATT
CTAAAATATGCTAGTGAAATTGAGATTGGGAATGTGGATTGTGACTTGCTGGAGCTTGCAATAAGCATGCTCATC
ACAAAGCTCAAGGCGTTAGGATTCCCAACTGTGAACTACATCACTGACCCAGAGGAAATTTTTAGTGCATTGAAT
ATGAAAGCAGCTATGGGAGCACTATACAAAGGCAAGAAGAAAGAAGCTCTCAGCGAGCTCACACTAGATGAGCAG
GAGGCAATGCTCAAAGCAAGTTGCCTGCGACTGTATACGGGAAAGCTGGGAATTTGGAATGGCTCATTGAAAGCA
GAGTTGCGTCCAATTGAGAAGGTTGAAAACAACAAAACGCGAACTTTCACAGCAGCACCAATAGACACTCTTCTT
GCTGGTAAAGTTTGCGTGGATGATTTCAACAATCAATTTTATGATCTCAACATAAAGGCACCATGGACAGTTGGT
ATGACTAAGTTTTATCAGGGGTGGAATGAATTGATGGAGGCTTTACCAAGTGGGTGGGTGTATTGTGACGCTGAT
GGTTCGCAATTCGACAGTTCCTTGACTCCATTCCTCATTAATGCTGTATTGAAAGTGCGACTTGCCTTCATGGAG
GAATGGGATATTGGTGAGCAAATGCTGCGAAATTTGTACACTGAGATAGTGTATACACCAATCCTCACACCGGAT
GGTACTATCATTAAGAAGCATAAAGGCAACAATAGCGGGCAACCTTCAACAGTGGTGGACAACACACTCATGGTC
ATTATTGCAATGTTATACACATGTGAGAAGTGTGGAATCAACAAGGAAGAGATTGTGTATTACGTCAATGGCGAT
GACCTATTGATTGCCATTCACCCAGATAAAGCTGAGAGGTTGAGTGGATTCAAAGAATCTTTCGGAGAGTTGGGC
CTGAAATATGAATTTGACTGCACCACCAGGGACAAGACACAGTTGTGGTTCATGTCACACAGGGCTTTGGAGAGG
GATGGCATGTATATACCAAAGCTAGAAGAAGAAAGGATTGTTTCTATTTTGGAATGGGACAGATCCAAAGAGCCG
TCACATAGGCTTGAAGCCATCTGTGCATCAATGATCGAAGCATGGGGTTATGACAAGCTGGTTGAAGAAATCCGC
AATTTCTATGCATGGGTTTTGGAACAAGCGCCGTATTCACAGCTTGCAGAAGAAGGAAAGGCGCCATATCTGGCT
GAGACTGCGCTTAAGTTTTTGTACACATCTCAGCACGGAACAAACTCTGAGATAGAAGAGTATTTAAAAGTGTTG
TATGATTACGATATTCCAACGACTGAGAATCTTTATTTTCAGAGTGGCACTGTGGGTGCTGGTGTTGACGCTGGT

```
AAGAAGAAAGATCAAAAGGATGATAAAGTCGCTGAGCAGGCTTCAAAGGATAGGGATGTTAATGCTGGAACTTCA
GGAACATTCTCAGTTCCACGAATAAATGCTATGGCCACAAAACTTCAATATCCAAGGATGAGGGGAGAGGTGGTT
GTAAACTTGAATCACCTTTTAGGATACAAGCCACAGCAAATTGATTTGTCAAATGCTCGAGCCACACATGAGCAG
TTTGCCGCGTGGCATCAGGCAGTGATGACAGCCTATGGAGTGAATGAAGAGCAAATGAAAATATTGCTAAATGGA
TTTATGGTGTGGTGCATAGAAAATGGGACTTCCCCAAATTTGAACGGAACTTGGGTTATGATGGATGGTGAGGAG
CAAGTTTCATACCCGCTGAAACCAATGGTTGAAAACGCGCAGCCAACACTGAGGCAAATTATGACACACTTCAGT
GACCTGGCTGAAGCGTATATTGAGATGAGGAATAGGGAGCGACCATACATGCCTAGGTATGGTCTACAGAGAAAC
ATTACAGACATGAGTTTGTCACGCTATGCGTTCGACTTCTATGAGCTAACTTCAAAAACACCTGTTAGAGCGAGG
GAGGCGCATATGCAAATGAAAGCTGCTGCAGTACGAAACAGTGGAACTAGGTTATTTGGTCTTGATGGCAACGTG
GGTACTGCAGAGGAAGACACTGAACGGCACACAGCGCACGATGTGAACCGTAACATGCACACACTATTAGGGGTC
CGCCAGTGATAGTTTCTGCGTGTCTTTGCTTTCCGCTTTTAAGCTTATTGTAATATATATGAATAGCTATTCACA
GTGGGACTTGGTCTTGTGTTGAATGGTATCTTATATGTTTTAATATGTCTTATTAGTCTCATTACTTAGGCGAAC
GACAAAGTGAGGTCACCTCGGTCTAATTCTCCTATGTAGTGCGAGAAAAAAAAAAAAAAAAAAAAAAAAAAAAAA
AAAAAAAAAAAAAA
```

TEV sequence corresponds to Genbank accession number DQ986288 including two silent mutations (G273A and A1119G, in red). Limits between TEV cistrons are marked on blue background.

>TEV-crtB (insert between possitions 8517-8518 of TEV-wt)

```
TCAGGTACAATGAATAATCCGTCGTTACTCAATCATGCGGTCGAAACGATGGCAGTTGGCTCGAAAAGTTTTGCG
ACAGCCTCAAAGTTATTTGATGCAAAAACCCGGCGCAGCGTACTGATGCTCTACGCCTGGTGCCGCCATTGTGAC
GATGTTATTGACGATCAGACGCTGGGCTTTCAGGCCCGGCAGCCTGCCTTACAAACGCCCGAACAACGTCTGATG
CAACTTGAGATGAAAACGCGCCAGGCCTATGCAGGATCGCAGATGCACGAACCGGCGTTTGCGGCTTTTCAGGAA
GTGGCTATGGCTCATGATATCGCCCCGGCTTACGCGTTTGATCATCTGGAAGGCTTCGCCATGGATGTACGCGAA
GCGCAATACAGCCAACTGGATGATACGCTGCGCTATTGCTATCACGTTGCAGGCGTTGTCGGCTTGATGATGGCG
CAAATCATGGGCGTGCGGGATAACGCCACGCTGGACCGCGCCTGTGACCTTGGGCTGGCATTTCAGTTGACCAAT
ATTGCTCGCGATATTGTGGACGATGCGCATGCGGGCCGCTGTTATCTGCCGGCAAGCTGGCTGGAGCATGAAGGT
CTGAACAAAGAGAATTATGCGGCACCTGAAAACCGTCAGGCGCTGAGCCGTATCGCCCGTCGTTTGGTGCAGGAA
GCAGAACCTTACTATTTGTCTGCCACAGCCGGCCTGGCAGGGTTGCCCCTGCGTTCCGCCTGGGCAATCGCTACG
GCGAAGCAGGTTTACCGGAAAATAGGTGTCAAAGTTGAACAGGCCGGTCAGCAAGCCTGGGATCAGCGGCAGTCA
ACGACCACGCCCGAAAAATTAACGCTGCTGCTGGCCGCCTCTGGTCAGGCCCTTACTTCCCGGATGCGGGCTCAT
CCTCCCCGCCCTGCGCATCTCTGGCAGCGCCCGCTCACTACAGAGAACCTCTACTTTCAA
```

cDNA corresponding to *P. ananatis crtB* is on yellow background. Sequences corresponding to artificial TEV NIaPro cleavage sites are in blue.

>TEV-Ros1 (insert between possitions 8517-8518 of TEV-wt)

```
TCAGGTACAATGGAAAAGAATTGTCGTGGAGTGAGAAAAGGTACTTGGACCAAAGAAGAAGACACTCTCTTGAGG
CAATGTATAGAAGAGTATGGTGAAGGGAAATGGCATCAAGTTCCACACAGAGCAGGGTTGAACCGGTGTAGGAAG
AGTTGCAGGCTGAGGTGGTTGAATTATCTGAGGCCAAATATCAAAAGAGGTCGGTTTTCGAGAGATGAAGTGGAC
CTAATTGTGAGGCTTCATAAGCTGTTGGGTAACAAATGGTCGCTGATTGCTGGTAGAATTCCTGGAAGGACAGCT
AATGACGTGAAGAACTTTTGGAATACTCATGTGGGGAAGAATTTAGGCGAGGATGGAGAACGATGCCGGAAAAAT
GTTATGAACACAAAAACCATTAAGCTGACTAATATCGTAAGACCCCGAGCTCGGACCTTCACCGGATTGCACGTT
ACTTGGCCGAGAGAAGTCGGAAAAACCGATGAATTTTCAAATGTCCGGTTAACAACTGATGAGATTCCAGATTGT
GAGAAGCAAACGCAATTTTACAATGATGTTGCGTCGCCACAAGATGAAGTTGAAGACTGCATTCAGTGGTGGAGT
AAGTTGCTAGAAACAACGGAGGATGGGGAATTAGGAAACCTATTCGAGGAGGCCCAACAAATTGGAAATACTACA
GAGAACCTCTACTTTCAA
```

cDNA corresponding to *A.majus Ros1* is on red background. Sequences corresponding to artificial TEV NIaPro cleavage sites are in blue.

>TMV-crtB

```
GTATTTTTACAACAATTACCAACAACAACAAACAACAGACAACATTACAATTACTATTTACAATTACAATGGCAT
ACACACAGACAGCTACCACATCAGCTTTGCTGGACACTGTCCGAGGAAACAACTCCTTGGTCAATGATCTAGCAA
AGCGTCGTCTTTACGACACAGCGGTTGAAGAGTTTAACGCTCGTGACCGCAGGCCCAAGGTGAACTTTTCAAAAG
TAATAAGCGAGGAGCAGACGCTTATTGCTACCCGGGCGTATCCAGAATTCCAAATTACATTTTATAACACGCAAA
ATGCCGTGCATTCGCTTGCAGGTGGATTGCGATCTTTAGAACTGGAATATCTGATGATGCAAATTCCCTACGGAT
```

CATTGACTTATGACATAGGCGGGAATTTTGCATCGCATCTGTTCAAGGGACGAGCATATGTACACTGCTGCATGC
CCAACCTGGACGTTCGAGACATCATGCGGCACGAAGGCCAGAAAGACAGTATTGAACTATACCTTTCTAGGCTAG
AGAGAGGGGGGAAAACAGTCCCCAACTTCCAAAAGGAAGCATTTGACAGATACGCAGAAATTCCTGAAGACGCTG
TCTGTCACAATACTTTCCAGACATGCGAACATCAGCCGATGCAGCAATCAGGCAGAGTGTATGCCATTGCGCTAC
ACAGCATATATGACATACCAGCCGATGAGTTCGGGGCGGCACTCTTGAGGAAAAATGTCCATACGTGCTATGCCG
CTTTCCACTTCTCCGAGAACCTGCTTCTTGAAGATTCATGCGTCAATTTGGACGAAATCAACGCGTGTTTTTCGC
GCGATGGAGACAAGTTGACCTTTTCTTTTGCATCAGAGAGTACTCTTAATTACTGTCATAGTTATTCTAATATTC
TTAAGTATGTGTGCAAAACTTACTTCCCGGCCTCTAATAGAGAGGTTTACATGAAGGAGTTTTTAGTCACCAGAG
TTAATACCTGGTTTTGTAAGTTTTCTAGAATAGATACTTTTCTTTTGTACAAAGGTGTGGCCCATAAAAGTGTAG
ATAGTGAGCAGTTTTATACTGCAATGGAAGACGCATGGCATTACAAAAAGACTCTTGCAATGTGCAACAGCGAGA
GAATCCTCCTTGAGGATTCATCATCAGTCAATTACTGGTTTCCCAAAATGAGGGATATGGTCATCGTACCATTAT
TCGACATTTCTTTGGAGACTAGTAAGAGGACGCGCAAGGAAGTCTTAGTGTCCAAGGATTTCGTGTTTACAGTGC
TTAACCACATTCGAACATACCAGGCGAAAGCTCTTACATACGCAAATGTTTTGTCCTTCGTCGAATCGATTCGAT
CGAGGGTAATCATTAACGGTGTGACAGCGAGGTCCGAATGGATGTGGACAAATCTTTGTTACAATCCTTGTCCA
TGACGTTTTACCTGCATACTAAGCTTGCCGTTCTAAAGGATGACTTACTGATTAGCAAGTTTAGTCTCGGTTCGA
AAACGGTGTGCCAGCATGTGTGGGATGAGATTTCGCTGGCGTTTGGGAACGCATTTCCCTCCGTGAAAGAGAGGC
TCTTGAACAGGAAACTTATCAGAGTGGCAGGCGACGCATTAGAGATCAGGGTGCCTGATCTATATGTGACCTTCC
ACGACAGATTAGTGACTGAGTACAAGGCCTCTGTGGACATGCCTGCGCTTGACATTAGGAAGAAGATGGAAGAAA
CGGAAGTGATGTACAATGCACTTTCAGAATTATCGGTGTTAAGGGAGTCTGACAAATTCGATGTTGATGTTTTTT
CCCAGATGTGCCAATCTTTGGAAGTTGACCCAATGACGGCAGCGAAGGTTATAGTCGCGGTCATGAGCAATGAGA
GCGGTCTGACTCTCACATTTGAACGACCTACTGAGGCGAATGTTGCGCTAGCTTTACAGGATCAAGAGAAGGCTT
CAGAAGGTGCATTGGTAGTTACCTCAAGAGAAGTTGAAGAACCGTCCATGAAGGGTTCGATGGCCAGAGGAGAGT
TACAATTAGCTGGTCTTGCTGGAGATCATCCGGAATCGTCCTATTCTAAGAACGAGGAGATAGAGTCTTTAGAGC
AGTTTCATATGGCGACGGCAGATTCGTTAATTCGTAAGCAGATGAGCTCGATTGTGTACACGGGTCCGATTAAAG
TTCAGCAAATGAAAAACTTTATCGATAGCCTGGTAGCATCACTATCTGCTGCGGTGTCGAATCTCGTCAAGATCC
TCAAAGATACAGCTGCTATTGACCTTGAAACCCGTCAAAAGTTTGGAGTCTTGGATGTTGCATCTAGGAAGTGGT
TAATCAAACCAACGGCCAAGAGTCATGCATGGGGTGTTGTTGAAACCCACGCGAGGAAGTATCATGTGGCGCTTT
TGGAATATGATGAGCAGGGTGTGGTGACATGCGATGATTGGAGAAGAGTAGCTGTTAGCTCTGAGTCTGTTGTTT
ATTCCGACATGGCGAAACTCAGAACTCTGCGCAGACTGCTTCGAAACGGAGAACCGCATGTCAGTAGCGCAAAGG
TTGTTCTTGTGGACGGAGTTCCGGGCTGTGGAAAAACCAAAGAAATTCTTTCCAGGGTTAATTTTGATGAAGATC
TAATTTTAGTACCTGGGAAGCAAGCCGCGGAAATGATCAGAAGACGTGCGAATTCCTCAGGGATTATTGTGGCCA
CGAAGGACAACGTTAAAACCGTTGATTCTTTCATGATGAATTTTGGGAAAAGCACACGCTGTCAGTTCAAGAGGT
TATTCATTGATGAAGGGTTGATGTTGCATACTGGTTGTGTTAATTTTCTTGTGGCGATGTCATTGTGCGAAATTG
CATATGTTTACGGAGACACACAGCAGATTCCATACATCAATAGAGTTTCAGGATTCCCGTACCCCGCCCATTTTG
CCAAATTGGAAGTTGACGAGGTGGAGACACGCAGAACTACTCTCCGTTGTCCAGCCGATGTCACACATTATCTGA
ACAGGAGATATGAGGGCTTTGTCATGAGCACTTCTTCGGTTAAAAAGTCTGTTTCGCAGGAGATGGTCGGCGGAG
CCGCCGTGATCAATCCGATCTCAAAACCCTTGCATGGCAAGATCTTGACTTTTACCCAATCGGATAAAGAAGCTC
TGCTTTCAAGAGGGTATTCAGATGTTCACACTGTGCATGAAGTGCAAGGCGAGACATACTCTGATGTTTCACTAG
TTAGGTTAACCCCTACACCGGTCTCCATCATTGCAGGAGACAGCCCACATGTTTTGGTCGCATTGTCAAGGCACA
CCTGTTCGCTCAAGTACTACACTGTTGTTATGGATCCTTTAGTTAGTATCATTAGAGATCTAGAGAAACTTAGCT
CGTACTTGTTAGATATGTATAAGGTCGATGCAGGAACACAATAGCAATTACAGATTGACTCGGTGTTCAAAGGTT
CCAATCTTTTTGTTGCAGCGCCAAAGACTGGTGATATTTCTGATATGCAGTTTTACTATGATAAGTGTCTCCCAG
GCAACAGCACCATGATGAATAATTTTGATGCTGTTACCATGAGGTTGACTGACATTTCATTGAATGTCAAAGATT
GCATATTGGATATGTCTAAGTCTGTTGCTGCGCCTAAGGATCAAATCAAACCACTAATACCTATGGTACGAACGG
CGGCAGAAATGCCACGCCAGACTGGACTATTGGAAAATTTAGTGGCGATGATTAAAAGAAACTTTAACGCACCCG
AGTTGTCTGGCATCATTGATATTGAAAATACTGCATCTTTGGTTGTAGATAAGTTTTTTGATAGTTATTTGCTTA
AAGAAAAAAGAAAACCAAATAAAAATGTTTCTTTGTTCAGTAGAGAGTCTCTCAATAGATGGTTAGAAAAGCAGG
AACAGGTAACAATAGGCCAGCTCGCAGATTTTGATTTTGTGGATTTGCCAGCAGTTGATCAGTACAGACACATGA
TTAAAGCACAACCCAAACAAAAGTTGGACACTTCAATCCAAACGGAGTACCCGGCTTTGCAGACGATTGTGTACC
ATTCAAAAAAGATCAATGCAATATTCGGCCCGTTGTTTAGTGAGCTTACTAGGCAATTACTGGACAGTGTTGATT
CGAGCAGATTTTTGTTTTTCACAAGAAAGACACCAGCGCAGATTGAGGATTTCTTCGGAGATCTCGACAGTCATG
TGCCGATGGATGTCTTGGAGCTGGATATATCAAAATACGACAAATCTCAGAATGAATTCCACTGTGCAGTAGAAT
ACGAGATCTGGCGAAGATTGGGTTTCGAAGACTTCTTGGGAGAAGTTTGGAAACAAGGGCATAGAAAGACCACCC
TCAAGGATTATACCGCAGGTATAAAAACTTGCATCTGGTATCAAAGAAAGAGCGGGGACGTCACGACGTTCATTG
GAAACACTGTGATCATTGCTGCATGTTTGGCCTCGATGCTTCCGATGGAGAAAATAATCAAAGGAGCCTTTTGCG
GTGACGATAGTCTGCTGTACTTTCCAAAGGGTTGTGAGTTTCCGGATGTGCAACACTCCGCGAATCTTATGTGGA
ATTTTGAAGCAAAACTGTTTAAAAAACAGTATGGATACTTTTGCGGAAGATATGTAATACATCACGACAGAGGAT
GCATTGTGTATTACGATCCCCTAAAGTTGATCTCGAAACTTGGTGCTAAACACATCAAGGATTGGGAACACTTGG
AGGAGTTCAGAAGGTCTCTTTGTGATGTTGCTGTTTCGTTGAACAATTGTGCGTATTACACACAGTTGGACGACG
CTGTATGGGAGGTTCATAAGACCGCCCCTCCAGGTTCGTTTGTTTATAAAAGTCTGGTGAAGTATTTGTCTGATA
AAGTTCTTTTTAGAAGTTTGTTTATAGATGGCTCTAGTTGTTAAAGGAAAAGTGAATATCAATGAGTTTATCGAC

CTGACAAAAATGGAGAAGATCTTACCGTCGATGTTTACCCCTGTAAAGAGTGTTATGTGTTCCAAAGTTGATAAA
ATAATGGTTCATGAGAATGAGTCATTGTCAGGGGTGAACCTTCTTAAAGGAGTTAAGCTTATTGATAGTGGATAC
GTCTGTTTAGCCGGTTTGGTCGTCACGGGCGAGTGGAACTTGCCTGACAATTGCAGAGGAGGTGTGAGCGTGTGT
CTGGTGGACAAAAGGATGGAAAGAGCCGACGAGGCCAtTCTCGGATCTTACTACACAGCAGCTGCAAAGAAAAGA
TTTCAGTTCAAGGTCGTTCCCAATTATGCTATAACCACCCAGGACGCGATGAAAAACGTCTGGCAAGTTTTAGTT
AATATTAGAAATGTGAAGATGTCAGCGGGTTTCTGTCCGCTTTCTCTGGAGTTTGTGTCGGTGTGTATTGTTTAT
AaAAATAATATAAAATTAGGTTTGAGAGAGAAGATTACAAACGTGAGAGACGGAGGGCCCATGGAACTTACAGAA
GAAGTCGTTGATGAGTTCATGGAAGATGTCCCTATGTCGATCAGGCTTGCAAAGTTTCGATCTCGAACCGGAAAA
AAGAGTGATGTCCGCAAAGGGAAAAATAGTAGTAGTGATCGGTCAGTGCCGAACAAGAACTATAGAAATGTTAAG
GATTTTGGAGGAATGAGTTTTAAAAAGAATAATTTAATCGATGATGATTCGGAGGCTACTGTCGCCGAATCGGAT
TCGTTTTAAATAGATCTTACAGTATCACTACTCCATCTCAGTTCGTGTTCTTGTCATTAATATGAATAATCCGTC
GTTACTCAATCATGCGGTCGAAACGATGGCAGTTGGCTCGAAAAGTTTTGCGACAGCCTCAAAGTTATTTGATGC
AAAAACCCGGCGCAGCGTACTGATGCTCTACGCCTGGTGCCGCCATTGTGACGATGTTATTGACGATCAGACGCT
GGGCTTTCAGGCCCGGCAGCCTGCCTTACAAACGCCCGAACAACGTCTGATGCAACTTGAGATGAAAACGCGCCA
GGCCTATGCAGGATCGCAGATGCACGAACCGGCGTTTGCGGCTTTTCAGGAAGTGGCTATGGCTCATGATATCGC
CCCGGCTTACGCGTTTGATCATCTGGAAGGCTTCGCCATGGATGTACGCGAAGCGCAATACAGCCAACTGGATGA
TACGCTGCGCTATTGCTATCACGTTGCAGGCGTTGTCGGCTTGATGATGGCGCAAATCATGGGCGTGCGGATAA
CGCCACGCTGGACCGCGCCTGTGACCTTGGGCTGGCATTTCAGTTGACCAATATTGCTCGCGATATTGTGGACGA
TGCGCATGCGGGCCGCTGTTATCTGCCGGCAAGCTGGCTGGAGCATGAAGGTCTGAACAAAGAGAATTATGCGGC
ACCTGAAAACCGTCAGGCGCTGAGCCGTATCGCCCGTCGTTTGGTGCAGGAAGCAGAACCTTACTATTTGTCTGC
CACAGCCGGCCTGGCAGGGTTGCCCCTGCGTTCCGCCTGGGCAATCGCTACGGCGAAGCAGGTTTACCGGAAAAT
AGGTGTCAAAGTTGAACAGGCCGGTCAGCAAGCCTGGGATCAGCGGCAGTCAACGACCACGCCCGAAAAATTAAC
GCTGCTGCTGGCCGCCTCTGGTCAGGCCCTTACTTCCCGGATGCGGGCTCATCCTCCCCGCCCTGCGCATCTCTG
GCAGCGCCCGCTCTAAGGGGTAGTCAAGATGCATAATAAATAACGGATTGTGTCCGTAATCACACGTGGTGCGTA
CGATAACGCATAGTGTTTTTCCCTCCACTTAAATCGAAGGGTTGTGTCTTGGATCGCGCGGGTCAAATGTATATG
GTTCATATACATCCGCAGGCACGTAATAAAGCGAGGGGTTCGGGTCGAGGTCGGCTGTGAAACTCGAAAAGGTTC
CGGAAAACAAAAAAGAGAGTGGTAGGTAATAGTGTTAATAATAAGAAAATAAATAATAGTGGTAAGAAAGGTTTG
AAAGTTGAGGAAATTGAGGATAATGTAAGTGATGACGAGTCTATCGCGTCATCGAGTACGTTTTAATCAATATGC
CTTATACAATCAACTCTCCGAGCCAATTTGTTTACTTAAGTTCCGCTTATGCAGATCCTGTGCAGCTGATCAATC
TGTGTACAAATGCATTGGGTAACCAGTTTCAAACGCAACAAGCTAGGACAACAGTCCAACAGCAATTTGCGGATG
CCTGGAAACCTGTGCCTAGTATGACAGTGAGATTTCCTGCATCGGATTTCTATGTGTATAGATATAATTCGACGC
TTGATCCGTTGATCACGGCGTTATTAAATAGCTTCGATACTAGAAATAGAATAATAGAGGTTGATAATCAACCCG
CACCGAATACTACTGAAATCGTTAACGCGACTCAGAGGGTAGACGATGCGACTGTAGCTATAAGGGCTTCAATCA
ATAATTTGGCTAATGAACTGGTTCGTGGAACTGGCATGTTCAATCAAGCAAGCTTTGAGACTGCTAGTGGACTTG
TCTGGACCACAACTCCGGCTACTTAGCTATTGTTGTGAGATTTCCTAAAATAAAGTCACTGAAGACTTAAAATTC
AGGGTGGCTGATACCAAAATCAGCAGTGGTTGTTCGTCCACTTAAATATAACGATTGTCATATCTGGATCCAACA
GTTAAACCATGTGATGGTGTATACTGTGGTATGGCGTAAAACAACGGAAAAGTCGCTGAAGACTTAAAATTCAGG
GTGGCTGATACCAAAATCAGCAGTGGTTGTTCGTCCACTTAAAAATAACGATTGTCATATCTGGATCCAACAGTT
AAACCATGTGATGGTGTATACTGTGGTATGGCGTAAAACAACGGAGAGGTTCGAATCCTCCCCTAACCGCGGGTA
GCGGCCCA

TMV sequence corresponds to vector TMV 30B, which includes the 3’ end of *Tobacco mild green mosaic virus* (see reference 41). cDNA corresponding to *P. ananatis crtB* is on yellow background.

>PVX-crtB
GAAAACTAAACCATACACCACCAACACAACCAAACCCACCACGCCCAATTGTTACACACCCGCTTGAAAAAGAAA
GTTTAACAAATGGCCAAGGTGCGCGAGGTTTACCAATCTTTTACAGACTCCACCACAAAAACTCTCATCCAAGAT
GAGGCTTATAGAAACATTCGCCCCATCATGGAAAAACACAAACTAGCTAACCCTTACGCTCAAACGGTTGAAGCG
GCTAATGATCTAGAGGGGTTCGGCATAGCCACCAATCCCTATAGCATTGAATTGCATACACATGCAGCCGCTAAG
ACCATAGAGAATAAACTTCTAGAGGTGCTTGGTTCCATCCTACCACAAGAACCTGTTACATTTATGTTTCTTAAA
CCCAGAAAGCTAAACTACATGAGAAGAAACCCGCGGATCAAGGACATTTTCCAAAATGTTGCCATTGAACCAAGA
GACGTAGCCAGGTACCCCAAGGAAACAATAATTGACAAACTCACAGAGATCACAACGGAAACAGCATACATTAGT
GACACTCTGCACTTCTTGGATCCGAGCTACATAGTGGAGACATTCCAAAACTGCCCAAAATTGCAAACATTGTAT
GCGACCTTAGTTCTCCCCGTTGAGGCAGCCTTTAAAATGGAAAGCACTCACCCGAACATATACAGCCTCAAATAC
TTCGGAGATGGTTTCCAGTATATACCAGGCAACCATGGTGGCGGGGCATACCATCATGAATTCGCTCATCTACAA
TGGCTCAAAGTGGGAAAGATCAAGTGGAGGGACCCCAAGGATAGCTTTCTCGGACATCTCAATTACACGACTGAG
CAGGTTGAGATGCACACAGTGACAGTACAGTTGCAGGAATCGTTCGCGGCAAACCACTTGTACTGCATCAGGAGA
GGAGACTTGCTCACACCGGAGGTGCGCACTTTCGGCCAACCTGACAGGTACGTGATTCCACCACAGATCTTCCTC
CCAAAAGTTCACAACTGCAAGAAGCCGATTCTCAAGAAAACTATGATGCAGCTCTTCTTGTATGTTAGGACAGTC

AAGGTCGCAAAAAATTGTGACATTTTTGCCAAAGTCAGACAATTAATTAAATCATCTGACTTGGACAAATACTCT
GCTGTGGAACTGGTTTACTTAGTAAGCTACATGGAGTTCCTTGCCGATTTACAAGCTACCACCTGCTTCTCAGAC
ACACTTTCTGGTGGCTTGCTAACAAAGACCCTTGCACCGGTGAGGGCTTGGATACAAGAGAAAAAGATGCAGCTG
TTTGGTCTTGAGGACTACGCGAAGTTAGTCAAAGCAGTTGATTTCCACCCGGTGGATTTTTCTTTCAAAGTGGAA
ACTTGGGACTTCAGATTCCACCCCTTGCAAGCGTGGAAAGCCTTCCGACCAAGGGAAGTGTCGGATGTAGAGGAA
ATGGAAAGTTTGTTCTCAGATGGGGACCTGCTTGATTGCTTCACAAGAATGCCAGCTTATGCGGTAAACGCAGAG
GAAGATTTAGCTGCAATCAGGAAAACGCCCGAGATGGATGTCGGTCAAGAAGTTAAAGAGCCTGCAGGAGACAGA
AATCAATACTCAAACCCTGCAGAAACTTTCCTCAACAAGCTCCACAGGAAACACAGTAGGGAGGTGAAACACCAG
GCCGCAAAGAAAGCTAAACGCCTAGCTGAAATCCAGGAGTCAATGAGAGCTGAAGGTGATGCCGAACCAAATGAA
ATAAGCGGGACGATGGGGGCAATACCCAGCAACGCCGAACTTCCTGGCACGAATGATGCCAGACAAGAACTCACA
CTCCCAACCACTAAACCTGTCCCTGCAAGGTGGGAAGATGCTTCATTCACAGATTCTAGTGTGGAAGAGGAGCAG
GTTAAACTCCTTGGAAAAGAAACCGTTGAAACAGCGACGCAACAAGTCATCGAAGGACTTCCTTGGAAACACTGG
ATTCCTCAATTAAATGCTGTTGGATTCAAGGCGCTGGAAATTCAGAGGGATAGGAGTGGAACAATGATCATGCCC
ATCACAGAAATGGTCTCCGGGCTGGAAAAAGAGGACTTCCCTGAAGGAACTCCAAAAGAGTTGGCACGAGAATTG
TTCGCTATGAACAGAAGCCCTGCCACCATCCCTTTGGACCTGCTTAGAGCCAGAGACTACGGCAGTGATGTAAAG
AACAAGAGAATTGGTGCCATCACAAAGACACAGGCAACGAGTTGGGGCGAATACTTGACAGGAAAGATAGAAAGC
TTAACTGAGAGGAAAGTTGCGACTTGTGTCATTCATGGAGCTGGAGGTTCTGGAAAAAGTCATGCCATCCAGAAG
GCATTGAGAGAAATTGGCAAGGGCTCGGACATCACTGTAGTCCTGCCGACCAATGAACTGCGGCTAGATTGGAGT
AAGAAAGTGCCTAACACTGAGCCCTATATGTTCAAGACCTCTGAAAAGGCGTTAATTGGGGGAACAGGCAGCATA
GTCATCTTTGACGATTACTCAAAACTTCCTCCCGGTTACATAGAAGCCTTAGTCTGTTTCTACTCTAAAATCAAG
CTAATCATTCTAACAGGAGATAGCAGACAAAGCGTCTACCATGAAACTGCTGAGGACGCCTCCATCAGGCATTTG
GGACCAGCAACAGAGTACTTCTCAAAATACTGCCGATACTATCTCAATGCCACACACCGCAACAAGAAAGATCTT
GCGAACATGCTTGGTGTCTACAGTGAGAGAACGGGAGTCACCGAAATCAGCATGAGCGCCGAGTTCTTAGAAGGA
ATCCCAACTTTGGTACCCTCGGATGAGAAGAGAAAGCTGTACATGGGCACCGGGAGGAATGACACGTTCACATAC
GCTGGATGCCAGGGGCTAACTAAGCCGAAGGTACAAATAGTGTTGGACCACAACACCCAAGTGTGTAGCGCGAAT
GTGATGTACACGGCACTTTCTAGAGCCACCGATAGGATTCACTTCGTGAACACAAGTGCAAATTCCTCTGCCTTC
TGGGAAAAGTTGGACAGCACCCCTTACCTCAAGACTTTCCTATCAGTGGTGAGAGAACAAGCACTCAGGGAGTAC
GAGCCGGCAGAGGCAGAGCCAATTCAAGAGCCTGAGCCCCAGACACACATGTGTGTCGAGAATGAGGAGTCCGTG
CTAGAAGAGTACAAAGAGGAACTCTTGGAAAAGTTTGACAGAGAGATCCACTCTGAATCCCATGGTCATTCAAAC
TGTGTCCAAACTGAAGACACAACCATTCAGTTGTTTTCGCATCAACAAGCAAAAGATGAGACCCTCCTCTGGGCG
ACTATAGATGCGCGGCTCAAGACCAGCAATCAAGAAACAAACTTCCGAGAATTCCTGAGCAAGAAGGACATTGGG
GACGTTCTGTTTTTAAACTACCAAAAAGCTATGGGTTTACCCAAAGAGCGTATTCCTTTTTCCCAAGAGGTCTGG
GAAGCTTGTGCCCACGAAGTACAAAGCAAGTACCTCAGCAAGTCAAAGTGCAACTTGATCAATGGGACTGTGAGA
CAGAGCCCAGACTTCGATGAAAATAAGATTATGGTATTCCTCAAGTCGCAGTGGGTCACAAAGGTGGAAAAACTA
GGTCTACCCAAGATTAAGCCAGGTCAAACCATAGCAGCCTTTTACCAGCAGACTGTGATGCTTTTTGGAACTATG
GCTAGGTACATGCGATGGTTCAGACAGGCTTTCCAGCCAAAAGAAGTCTTCATAAACTGTGAGACCACGCCAGAT
GACATGTCTGCATGGGCCTTGAACAACTGGAATTTCAGCAGACCTAGCTTGGCTAATGACTACACAGCTTTCGAC
CAGTCTCAGGATGGAGCCATGTTGCAATTTGAGGTGCTCAAAGCCAAACACCACTGCATACCAGAGGAAATCATT
CAGGCATACATAGATATTAAGACTAATGCACAGATTTTCCTAGGCACGTTATCAATTATGCGCCTGACTGGTGAA
GGTCCCACTTTTGATGCAAACACTGAGTGCAACATAGCTTACACCCATACAAAGTTTGACATCCCAGCCGGAACT
GCTCAAGTTTATGCAGGAGACGACTCCGCACTGGACTGTGTTCCAGAAGTGAAGCATAGTTTCCACAGGCTTGAG
GACAAATTACTCCTAAAGTCAAAGCCTGTAATCACGCAGCAAAAGAAGGGCAGTTGGCCTGAGTTTTGTGGTTGG
CTGATCACACCAAAAGGGGTGATGAAAGACCCAATTAAGCTCCATGTTAGCTTAAAATTGGCTGAAGCTAAGGGT
GAACTCAAGAAATGTCAAGATTCCTATGAAATTGATCTGAGTTATGCCTATGACCACAAGGACTCTCTGCATGAC
TTGTTCGATGAGAAACAGTGTCAGGCACACACACTCACTTGCAGAACACTAATCAAGTCAGGGAGAGGCACTGTC
TCACTTTCCCGCCTCAGAAACTTTCTTTAACCGTTAAGTTACCTTAGAGATTTGAATAAGATGGATATTCTCATC
AGTAGTTTGAAAAGTTTAGGTTATTCTAGGACTTCCAAATCTTTAGATTCAGGACCTTTGGTAGTACATGCAGTA
GCCGGAGCCGGTAAGTCCACAGCCCTAAGGAAGTTGATCCTCAGACACCCAACATTCACCGTGCATACACTCGGT
GTCCCTGACAAGGTGAGTATCAGAACTAGAGGCATACAGAAGCCAGGACCTATTCCTGAGGGCAACTTCGCAATC
CTCGATGAGTATACTTTGGACAACACCACAAGGAACTCTAACCAGGCACTTTTTGCTGACCCTTATCAGGCACCG
GAGTTTAGCCTAGAGCCCCACTTCTACTTGGAAACATCATTTCGAGTTCCGAGGAAAGTGGCAGATTTGATAGCT
GGCTGTGGCTTCGATTTCGAGACCAACTCACCGGAAGAAGGGCACTTAGAGATCACTGGCATATTCAAAGGGCCC
CTACTCGGAAAGGTGATAGCCATTGATGAGGAGTCTGAGACAACACTGTCCAGGCATGGTGTTGAGTTTGTTAAG
CCCTGCCAAGTGACGGGACTTGAGTTCAAAGTAGTCACTATTGTGTCTGCCGCACCAATAGAGGAAATTGGCCAG
TCCACAGCTTTCTACAACGCTATCACCAGGTCAAAGGGATTGACATATGTCCGCGCAGGGCCATAGGCTGACCGC
TCCGGTCAATTCTGAAAAAGTGTACATAGTATTAGGTCTATCATTTGCTTTAGTTTCAATTACCTTTCTGCTTTC
TAGAAATAGCTTACCCCACGTCGGTGACAACATTCACAGCTTGCCACACGGAGGAGCTTACAGAGACGGCACCAA
AGCAATCTTGTACAACTCCCCAAATCTAGGGTCACGAGTGAGTCTACACAACGGAAAGAACGCAGCATTTGCTGC
CGTTTTGCTACTGACTTTGCTGATCTATGGAAGTAAATACATATCTCAACGCAATCATACTTGTGCTTGTGGTAA
CAATCATAGCAGTCATTAGCACTTCCTTAGTGAGGACTGAACCTTGTGTCATCAAGATTACTGGGGAATCAATCA
CAGTGTTGGCTTGCAAACTAGATGCAGAAACCATAAGGGCCATTGCCGATCTCAAGCCACTCTCCGTTGAACGGT

TAAGTTTCCATTGATACTCGAAAGAGGTCAGCACCAGCTAGCATGAATAATCCGTCGTTACTCAATCATGCGGTC
GAAACGATGGCAGTTGGCTCGAAAAGTTTTGCGACAGCCTCAAAGTTATTTGATGCAAAAACCCGGCGCAGCGTA
CTGATGCTCTACGCCTGGTGCCGCCATTGTGACGATGTTATTGACGATCAGACGCTGGGCTTTCAGGCCCGGCAG
CCTGCCTTACAAACGCCCGAACAACGTCTGATGCAACTTGAGATGAAAACGCGCCAGGCCTATGCAGGATCGCAG
ATGCACGAACCGGCGTTTGCGGCTTTTCAGGAAGTGGCTATGGCTCATGATATCGCCCCGGCTTACGCGTTTGAT
CATCTGGAAGGCTTCGCCATGGATGTACGCGAAGCGCAATACAGCCAACTGGATGATACGCTGCGCTATTGCTAT
CACGTTGCAGGCGTTGTCGGCTTGATGATGGCGCAAATCATGGGCGTGCGGGATAACGCCACGCTGGACCGCGCC
TGTGACCTTGGGCTGGCATTTCAGTTGACCAATATTGCTCGCGATATTGTGGACGATGCGCATGCGGGCCGCTGT
TATCTGCCGGCAAGCTGGCTGGAGCATGAAGGTCTGAACAAAGAGAATTATGCGGCACCTGAAAACCGTCAGGCG
CTGAGCCGTATCGCCCGTCGTTTGGTGCAGGAAGCAGAACCTTACTATTTGTCTGCCACAGCCGGCCTGGCAGGG
TTGCCCCTGCGTTCCGCCTGGGCAATCGCTACGGCGAAGCAGGTTTACCGGAAAATAGGTGTCAAAGTTGAACAG
GCCGGTCAGCAAGCCTGGGATCAGCGGCAGTCAACGACCACGCCCGAAAAATTAACGCTGCTGCTGGCCGCCTCT
GGTCAGGCCCTTACTTCCCGGATGCGGGCTCATCCTCCCCGCCCTGCGCATCTCTGGCAGCGCCCGCTCTAGGGT
TTGTTAAGTTTCCCTTTTTACTCGAAAGATGTCAGGCCTGTTCACCATCCCGGATGGGGATTTCTTTAGTACAGC
CCGTGCCATAGTAGCCAGCAATGCTGTCGCAACAAATGAGGACCTCAGCAAGATTGAGGCTATTTGGAAGGACAT
GAAGGTGCCCACAGACACTATGGCACAGGCTGCTTGGGACTTAGTCAGACACTGTGCTGATGTAGGATCATCCGC
TCAAACAGAAATGATAGATACAGGTCCCTATTCCAACGGCATCAGCAGAGCTAGACTGGCAGCAGCAATTAAAGA
GGTGTGCACACTTAGGCAATTTTGCATGAAGTATGCTCCAGTGGTATGGAACTGGATGTTAACTAACAACAGTCC
ACCTGCTAACTGGCAAGCACAAGGTTTCAAGCCTGAGCACAAATTCGCTGCATTCGACTTCTTCAATGGAGTCAC
CAACCCAGCTGCCATCATGCCCAAAGAGGGGCTCATCCGGCCACCGTCTGAAGCTGAAATGAATGCTGCCCAAAC
TGCTGCCTTTGTGAAGATTACAAAGGCCAGGGCACAATCCAACGACTTTGCCAGCCTAGATGCAGCTGTCACTCG
AGGTCGTATCACTGGAACAACAACCGCTGAGGCTGTTGTCACTCTACCACCACCATAACTACGTCTACATAACCG
ACGCCTACCCCAGTTTCATAGTATTTTCTGGTTTGATTGTATGAATAATATAAATAAAAAAAAAAAAAAAAAAAA
AAAAAAAAAAAAAAAAAAAAAAAAAAAAAAAAAAAAAAAAAAAAAAAAAAAAAAAAAAAAAAAAAA

PVX sequence corresponds to M95516.1, but contains a heterologous promoter that derives from *Bamboo mosaic virus* and the deletion of the 29 initial codons of PVX coat protein (see reference 44). cDNA corresponding to *P. ananatis crtB* is on yellow background.

>ZYMV-wt
AAATTAAAACAAATCACAAAGACTACAGAAATCAACGAACAAACAAACGAATTTTAAACCGTGTTAACAAACAAG
CAATTTATAATTCGCACAGCATCAAGAATTTCTGCAATCATTTTGTTTATTTTAGACACAACAATGGCCTCAGTT
ATGATTGGTTCAATCTCCGTACCCATCGCACAGCCTGCGCAGTGTGCAAACACCCAAGCGAGCAACCGGGTTAAC
GTAGTGGCACCTGGCCACATGGCAACATGCCCACCATCATTGAAAACGCACACATACTACATGCATGAGTCTAAG
AAGTTGATAAATTCAGATAAAAGCAATGAAATTTTGAACAATTTCTTTAACACTGATGAGATGAAATTCGCGCTC
ACTAGGAATGAAATGAGTAAGGTGAAAAAGGGTCCGAATGGGAGGGTAGTTCTTCGCAAACCGAGCAAGCAGCGG
GTTTTCGCTCGGATCGAGCAGGATGAGGCAGCACGCAAGGAAGAAGCTGTTTTCCTTGCAGGAAATTATGATGGT
TCGATCACAAATCTAGTGAGTGTTCTTCCATCTGAAATGACTCGCGATGTTGATGCGAGTTTGCGATCACCATTT
TACAAGCGCACATATAAGAAGGACAGAAAGAAAGTGGCGCAAAAGCAAATCGCGCAGGCACCACTTAACAGCTTG
TGCACACGTGTTCTTAAAATTGCGCGCAATAGAAACATCCCTGTTGAGATAATTGGCAACAAGAAAGCAAGACAT
ACACTTACCTTCAAGAGGTTTAGGGGATGTTTTGTTGGAAAGGTGTCAGTTGCACATGAGGAAGGACGAATGCAA
CACACTGAGATATCATACGAGCAGTTTGAATGGATTCTACAAGCTATTTGTCGGGTTACTTATACAGAGCGAATT
CGTGAGGAAGACATTAAACCAGGTTGTAGTGGGTGGGTGTTAGGCACTGATCATACATTGACCAAGAGATATTCA
AGATTGCCACATCTGGTAATTCGAGGTAGAGATGATGACGGGATTGTGAACGCGCTGGAACCGGTGTTATTTTAC
AGCGAAGTTGACCACTATTCGTCGCAACCGGAAGTTCAGTTCTTCCAAGGATGGCGACGAATGTTCGACAAGTTT
AGACCCAGTCCAGATCATGTGTGCAAAGTTGATCACAACAATGAGGAGTGTGGTGAGTTAGCAGCAATCTTTTGT
CAGGCTTTGTTCCCAGTAGTGAAATTATCGTGCCAAACATGCAGAGAAAAGCTTAGTAGAGTTAGCTTCGAGGAA
TTCAAAGATTCTTTGAATACAAACTTTATTATCCATAAGGACGAATGGGATAGTTTCAAAGAGGGCTCTCATTAC
GATAATATTTTCAAATTAATTAAAGTAGCAACACAGGTAACTCAGAATCTCAAGCTCTCATCTGAAGTAATGAAG
TTAGTTCAGAACCACACAAGCACTCACATGAAGCAAATACAAGATATCAACAAGGCGCTCATGAAAGGTTCATTG
GTTACGCAAGACGAATTGGACTTGGCTTTGAAACAGCTTCTAGAAATGACTCAGTGGTTTAAGAACCACATGCAC
CTGACTGGTGAAGAAGCATTGAAGATGTTCAGAAACAAGCGCTCTAGCAAGGCTATGATAAATCCTAACCTTTTA
TGTGATAACCAGTTGGACAAAAATGGAAACTTTGTCTGGGGAGAAAGAGGATATCATTCCAAGCGATTATTCAAG
AACTTCTTTGAGGAAGTTATACCAAGTGAAGGATATACGAAATACGTAGTGCGAAACTTCCCAAATGGTACTCGT
AAGTTGGCCATAGGCTCATTGATCGTACCACTCAACTTGGATAGGGCACGCACTGCACTCCTTGGAGAGAGTATT
GAGAAGAAGCCACTCACATCAGCATGTGTCTCCCAACAGAATGGAAATTATATACACTCATGCTGCTGTGTGACG
ATGGATGATGGAACTCCGATGTACTCAGAGCTTAAGAGCCCGACGAAGAGACATCTAGTTATAGGAGCTTCTGGT
GATCCAAAGTACATTGATTTACCAGCATCTGAGGCAGAACGCATGTATATAGCAAAGGAAGGTTATTGCTATCTT
AACATTTTTCTCGCAATGCTTGTGAATGTTAATGAGAACGAGGCAAAGGATTTCACCAAAATGATTCGTGATGTT
TTGATCCCTATGCTTGGGCAATGGCCTTCGTTGATGGATGTCGCGACTGCAGCATACATTTTAGGTGTATTCCAT

CCTGAAACGCGATGCGCTGAATTACCTAGGATTCTTGTTGACCATGCTACGCAAACCATGCATGTCATTGATTCA
TATGGATCATTAACTGTTGGTTATCACGTGCTCAAGGCCGGAACTGTCAATCATTTAATTCAATTTGCCTCAAAT
GATCTGCAGAGCGAGATGAAGCATTACAGAGTCGGCGGAACGCCAACACAGCGCATAAAACTTGAGGAGCAATTG
ATTAAAGGAATCTTCAAACCAAAACTTATGATGCAGCTCTTGCACGATGACCCATACATATTATTGCTTGGCATG
ATCTCACCCACCATTCTTGTGCACATGTATAGGATGCGTCATTTTGAGCGAGGTATTGAAATATGGATTAAGAGA
GATCATGAAATTGGAAAGATTTTCGTCATATTGGAACAGCTCACACGGAAGGTTGCTCTGGCTGAAGTTCTTGTT
GATCAGCTCGATTTGATAAGTGAAGCTTCACCACATTTACTTGAAATCATGAAGGGTTGTCAAGATAATCAAAGG
GCGTATGTACCTGCGCTGGATCTGTTAACGATACAAGTAGAGCGTGAGTTTTCAAATAAAGAACTTAAAACCAAT
GGCTATCCAGATTTGCAGCAAACGTTGTTTGATATGAGAGAAAAAATGTATGCAAAGCAGTTGCACAGTTCATGG
CAAGAGCTAAGCTTGCTGGAAAAATCTTGTGTAACCGTGCGATTGAAGCAATTCTCGATTTTTACGGAAAGAAAT
TTAATCCAGCGAGCAAAAGAAGGAAAGCGCACATCTTCGCTACAATTTGTTCACGAGTGCTTTATCACGACCCGA
GTACATGCGAAGAGCATTCGCGATGCAGGCGTGCGCAAGCTAAATGAGGCTCTCGTTGGAACTTGTAAGTTCTTT
TTCTCTTGTGGTTTCAAGATTTTTGCGCGGTGTTACAGCGACATTATATACCTTGTGAACGTGTGTTTGGTATTC
TCTTTGGTGTTACAAATGTCTAATACTGTGCGCAACATGATAGCGGCGACAAGGGAAGAAAAGGAGAGAGCGATG
GCAAATAAAGCTGATGAAAATGAAAGGACGTTAATGCACATGTACCACATTTTCAGTAAGAAGCAGGATGAAGCG
CCCATATATAACGACTTTCTTGAACATGTTCGCAATGTAAGACCAGATCTTGAGGAAACCCTCTTGTACATGGCT
GGTGCAGAGGTTGTTGCAACACAGGCCAAGTCAGCGGTTCAGATTCAGTTCGAGAAGATTATAGCTGTATTGGCG
CTGCTCACTATGTGCTTTGACGCCGAAAGAAGTGATGCCATTTTCAAGATTTTGACAAAGCTCAAAACAGTTTTT
GGCACGGTTGGAGAAACGGTCCGACTTCAAGGACTTGAAGATATTGAGAGCTTGGAAGACGATAAGAGACTCACA
ATTGACTTTGATATTAACACGAATGAGGCTCAATCGTCGACAACGTTTGATGTTCATTTTGATGATTGGTGGAAT
CGGCAGCTACAGCAAAATCGCACAGTTCCACATTATAGGACCACAGGTAAATTCCTCGAATTCACCAGAAGCACT
GCAGCTTTTGTGGCTAATGAAATAGCATCATCAAGTGAAGGAGAATTTTTAGTTAGAGGAGCAGTGGGTTCTGGA
AAATCAACGAGCTTGCCTGCGCATCTTTCCAAGAAGGGTAAAGTATTACTACTCGAACCCACACGCCCTTTGGCG
GAGAATGTCAGTAGACAGTTGGCAGGCGATCCTTTTTTCCAAAACGTCACACTCAGAATGAGAGGGCTAAATTGC
TTTGGTTCAAGTAACATTACAGTGATGACGAGTGGATTTGCTTTTCACTACTATGTTAATAATCCACACCAATTA
ATGGAATTTGACTTTATTATCATAGACGAATGCCATGTTACGGACAGTGCGACTATAGCTTTCAATTGCGCACTT
AAGGAGTATAATTTTGCTGGCAAATTGATTAAAGTATCTGCAACGCCGCCAGGGAGGGAGTGTGATTTCGATACG
CAATTCGCGGTGAAAGTCAAAACGGAGGACCACCTTTCATTCCATGCATTCGTTGGCGCACAGAAGACTGGTTCA
AACGCTGATATGGTTCAGCATGGCAATAACATACTTGTGTATGTTGCAAGTTATAACGAAGTAGACATGCTTTCC
AAATTACTCACTGAGCGACAATTTTCAGTGACGAAGGTAGATGGGCGAACAATGCAACTTGGGAAAACTACCATT
GAAACGCATGGCACTAGTCAAAAGCCTCATTTCATAGTAGCCACAAACATCATTGAGAATGGAGTGACGTTGGAT
GTCGAATGTGTTGTTGATTTTGGGCTAAAAGTGGTCGCAGAATTAGACAGCGAAAATCGGTGTGTGCGCTACAAC
AAGAAATCAGTTAGTTATGGAGAAAGGATTCAGCGGCTAGGGAGAGTGGGGAGATCTAAGCCTGGAACTGCATTG
CGTATAGGGCACACAGAAAAAGGCATCGAGAACATTCCCGAATTCATTGCCACAGAAGCAGCAGCCTTATCATTT
GCATATGGGCTTCCAGTCACCACGCATGGGGTTTCCACAAATATACTCGGAAAGTGCACAGTTAAACAGATGAGA
TGTGCTTTGAATTTTGAGTTAACTCCTTTCTTTACCACTCATTTAATTCGCCATGATGGTAGCATGCACCCACTG
ATACACGAAGAACTGAAACAATTTAAACTCAGGGACTCAGAAATGGTGCTCAACAAGGTTGCATTACCTCACCAA
TTTGTGAGTCAATGGTTGGATCAAAGTGAGTATGAACGCATTGGAGTGCACGTTCAATGCCATGAGAGCACACGC
ATACCTTTTTACACAAATGGAGTGCCTGATAAAGTCTATGAGAAAATTTGGAAGTGCATACAAGAAAACAAGAAT
GATGCGGTTTTTGGTAAGCTTTCAAGTGCTTGTTCGACTAAGGTTAGTTATACACTCAGCACTGATCCAGCAGCA
TTACCCAGAACTATTGCAATCATCGACCACCTGCTTGCCGAGGAAATGATGAAGCGGAATCACTTCGACACGATC
AGCTCAGCTGTAACGGGCTATTCATTTTCCCTCGCTGGAATTGCTGATTCTTTTAGGAAAAGGTATATGCGCGAT
TACACAGCGCATAACATTGCAATTCTCCAACAAGCACGTGCCCAGCTGCTCGAATTCAATAGTAAAAATGTGAAC
ATCAACAACCTGTCCGATCTGGAAGGAATTGGAGTTATTAAGTCGGTGGTGTTGCAAAGTAAGCAAGAGGTCAGC
AACTTCCTAGGACTTCGCGGTAAATGGGATGGGCGGAAATTTGCGAATGATGTGATATTGGCGATCATGACACTC
TTAGGAGGTGGATGGTTCATGTGGGAATACTTCACGAAAAAGATCAATGAACCCGTGCGCGTTGAAAGCAAGAAA
CGGCGATCTCAAAAGTTGAAATTCAGGGATGCGTACGATAGGAAAGTCGGACGTGAGATTTTTGGCGATGATGAC
ACAATTGGGCGCACTTTTGGCGAAGCTTACACGAAGAGAGGAAAGGTCAAAGGAAACAACAGCACAAAAGGAATG
GGACGGAAAACTCGCAATTTTGTGCATTTATATGGTGTGGAGCCTGAGAATTACAGCTTTATTAGATTTGTGGAC
CCTCTCACTGGCCATACATTGGACGAAAGCACCCATACAGACATTTCGTTAGTGCAGGAGGAGTTTGGAAGTATT
AGAGAGAAATTTCTGGAGAATGATTTAATCTCGAGGCAGTCTATTATTAACAAACCCGGTATTCAAGCATATTTT
ATGGGCAAGGGCACCGAAGAAGCACTCAAAGTTGATTTGACTCCTCATGTACCATTGCTTCTGTGCAGAAACACC
AATGCCATTGCGGGGTATCCAGAGAGAGAAAATGAGTTGAGACAAACCGGCACACCAGTTAAGGTTTCTTTTAAA
GACGTGCCAGAGAAAAACGAACATGTCGAGTTGGAGAGCAAATCCATCTACAAAGGAGTGCGCGATTACAATGGC
ATTTCAACAATCGTCTGTCAATTAACGAACGATTCTGATGGCCTCAAGGAGACTATGTATGGTATTGGCTACGGG
CCGATAATCATTACTAATGGGCACCTCTTCAGGAAAAACAATGGTACACTTCTAGTCAGGTCTTGGCATGGTGAA
TTCACTGTTAAAAATACCACAACGCTCAAAGTGCATTTCATAGAAGGGAAGGATGTTGTTTTAGTGCGTATGCCA
AAGGACTTTCCACCGTTCAAAAGCAACGCTTCTTTTAGAGCGCCAAAACGCGAGGAACGAGCATGCTTGGTTGGA
ACAAATTTTCAAGAGAAGAGTCTCCGCTCCACTGTTTCAGAATCTTCAATGACAATACCTGAAGGAACTGGCTCA
TATTGGATTCATTGGATTTCAACCAATGAAGGGGACTGCGGATTACCCATGGTTTCAACAACGGATGGTAAGATA
ATTGGAGTTCATGGTTTGGCTTCCACAGTCTCATCTAAGAATTATTTTGTCCCATTCACTGATGATTTTATAGCC

ACGCATTTGAGCAAGCTTGATGATCTCACATGGACTCAGCATTGGCTATGGCAACCTAGCAAAATCGCGTGGGGA
ACGCTCAACTTAGTTGATGAACAACCAGGGCCTGAATTTCGTATTTCAAATCTAGTCAAGGATTTGTTCACTTCT
GGTGTTGAAACACAGAGCAAGCGGGAAAGATGGGTCTACGAAAGCTGTGAAGGGAACCTTCGAGCTGTTGGAACT
GCGCAATCAGCGTTAGTCACCAAACATGTTGTCAAGGGCAAGTGTTCTTTCTTCGAAGAATATTTGCAAACACAC
GCAGAAGCGAGCGCCTATTTCAGACCCTTAATGGGAGAGTACCAGCCGAGCAAGTTGAACAAAGAGGCCTTCAAA
AAGGATTTCTTTAAATACAACAAACCCGTCACTGTTAATCAATTGGATCATGATAAATTTTTAGAAGCAGTGGAT
GGGGTTATACGTATGATGTGTGACTTTGAGTTCAATGAATGCCGATTCATTACAGATCCCGAGGAAATTTACAAC
TCTCTGAACATGAAAGCAGCAATTGGAGCCCAATATAGAGGAAAGAAGAAAGAATATTTTGAAGGGCTAGATGAT
TTTGATCGAGAGCGACTATTATTTCAAAGTTGTGAAAGGTTGTTTAATGGCTATAAAGGTTTGTGGAATGGATCT
TTAAAGGCTGAGCTCAGGCCGCTTGAGAAAGTCAGGGCTAACAAAACACGAACTTTTACAGCAGCGCCAATTGAT
ACATTGCTTGGAGCTAAAGTTTGCGTGGATGATTTCAATAATGAATTTTACAGCAAAAATCTCAAGTGTCCATGG
ACGGTTGGCATGACAAAATTTTATGGTGGTTGGGATAAATTGATGAGATCGTTACCTGATGGTTGGTTATACTGT
CATGCTGATGGATCACAGTTTGACAGTTCATTAACCCCAGCTCTATTGAATGCAGTTCTTATAATCAGGTCTTTT
TACATGGAAGATTGGTGGGTTGGCCAAGAGATGCTCGAAAATCTCTATGCTGAGATTGTGTACACTCCAATTCTT
GCTCCGGATGGAACAATTTTCAAGAAATTTAGAGGTAACAACAGTGGGCAACCCTCAACAGTGGTGGATAACACA
CTAATGGTTGTGATCTCTATTTACTATGCGTGCATGAAGTTTGGGTGGAATTGCGAGGAAATTGAGAATAAACTT
GTCTTCTTTGCAAATGGAGATGACCTGATACTTGCAGTCAGAGATGAAGACAGCGGCTTACTTGATAACATGTCA
TCCTCTTTTTCCGAACTTGGACTGAATTACGATTTTTCGGAACGCACGCACAAAAGAGAAGATCTTTGGTTCATG
TCCCACCAGGCAATGTTAATTGATGGAATGTACATCCCAAAACTTGAGAAAGAGAGAATTGTTTCAATTCTAGAA
TGGGATAGAAGTAAGGAAATAATGCACCGAACAGAGGCTATTTGCGCTGCAATGATTGAAGCATGGGGACACACC
GAGCTTTTACAAGAAATCAGAAAGTTTTACCTGTGGTTCGTTGAAAAGGAAGAAGTGCGAGAATTGGCTGCCCTC
GGAAAAGCTCCATACATAGCTGAGACAGCTCTTCGTAAGTTATACACTGACAAGGGAGCGGATACGAGTGAACTG
GCACGCTATCTACGAGCCCTCCATCAAGATATCTTCTTTGAACAAGGAGACACTGTAATGCTCCAATCAGGCACT
CAGCCAACTGTGGCAGACGCTGGGGCTACAAAGAAAGACAAAGAAGATGACAAAGGGAAAAACAAGGATGTTTCA
GGCTCCGGCTCAGGTGAGAAAACGATAGCAGCTGTCACAAAGGACAAGGATGTGAATGCTGGTTCTCATGGGAAG
ATCGTGCCGCGTCTTTCAAAGATCACAAAGAAAATGTCACTGCCACGCGTGAAAGGAAATGTGATACTCGATATC
GATCATTTGCTGGAATATAAACCGGATCAAATCGAGTTGTACAACACACGAGCGTCTCATCAGCAATTCGCTTCC
TGGTTTAACCAAGTTAAAACAGAATATGATCTGAATGAGCAACAGATGGGAGTTGTAATGAATGGTTTCATGGTT
TGGTGTATTGAAAATGGCACCTCACCCGACATTAATGGAGTGTGGGTTATGATGGACGGAAATGAGCAAGTTGAA
TATCCTTTGAAACCAATAGTTGAAAATGCAAAGCCAACGCTGCGGCAAATAATGCATCATTTTTCAGATGCAGCG
GAGGCATACATAGAGATGAGAAATGCAGAGGCACCATACATGCCGAGGTATGGTTTGCTTCGAAACCTACGGGAT
AGGAGTTTGGCACGATATGCTTTCGATTTCTATGAAGTCAATTCTAAAACTCCTGAAAGAGCCCGTGAAGCTGTT
GCGCAAATGAAAGCAGCAGCTCTTAGCAATGTTTCTTCAAGGTTGTTTGGCCTTGATGGAAATGTTGCCACTACT
AGCGAAGACACTGAACGGCACACTGCACGTGATGTTAATAGAAACATGCACACCTTACTAGGTGTGAATACAATG
CAGTAAAGGGTAGGTCGCCTACCTAGGTTATTGTTTCGCTGCCGACGTAATTCTAATATTTACCGCTTTATTTGA
TATCTTTAAATTTCTAGAGTGGGCTTCCCACCCTTAAAGCGTAAAGTTTATGTTAGTTGTCCAGGAGTGCCGTAG
TCCTGTCGGAAGCTTTAGTGTGAGCCTCTCACGAATAAGCTCGAGATTAGACTCCGTTTGCAAGCCTAAAAAAAA
AAAAAAAAAAAAAAAAAAAAAAAAAAAAAAAAAAAAAAAAA

This ZYMV sequence is pending to deposit in GenBank. Limits between ZYMV cistrons are marked on blue background.

>ZYMV-crtB (insert between possitions 8541-8542 of ZYMV-wt)
TCTGGCACAATGAATAATCCGTCGTTACTCAATCATGCGGTCGAAACGATGGCAGTTGGCTCGAAAAGTTTTGCG
ACAGCCTCAAAGTTATTTGATGCAAAAACCCGGCGCAGCGTACTGATGCTCTACGCCTGGTGCCGCCATTGTGAC
GATGTTATTGACGATCAGACGCTGGGCTTTCAGGCCCGGCAGCCTGCCTTACAAACGCCCGAACAACGTCTGATG
CAACTTGAGATGAAAACGCGCCAGGCCTATGCAGGATCGCAGATGCACGAACCGGCGTTTGCGGCTTTTCAGGAA
GTGGCTATGGCTCATGATATCGCCCCGGCTTACGCGTTTGATCATCTGGAAGGCTTCGCCATGGATGTACGCGAA
GCGCAATACAGCCAACTGGATGATACGCTGCGCTATTGCTATCACGTTGCAGGCGTTGTCGGCTTGATGATGGCG
CAAATCATGGGCGTGCGGGATAACGCCACGCTGGACCGCGCCTGTGACCTTGGGCTGGCATTTCAGTTGACCAAT
ATTGCTCGCGATATTGTGGACGATGCGCATGCGGGCCGCTGTTATCTGCCGGCAAGCTGGCTGGAGCATGAAGGT
CTGAACAAAGAGAATTATGCGGCACCTGAAAACCGTCAGGCGCTGAGCCGTATCGCCCGTCGTTTGGTGCAGGAA
GCAGAACCTTACTATTTGTCTGCCACAGCCGGCCTGGCAGGGTTGCCCCTGCGTTCCGCCTGGGCAATCGCTACG
GCGAAGCAGGTTTACCGGAAAATAGGTGTCAAAGTTGAACAGGCCGGTCAGCAAGCCTGGGATCAGCGGCAGTCA
ACGACCACGCCCGAAAAATTAACGCTGCTGCTGGCCGCCTCTGGTCAGGCCCTTACTTCCCGGATGCGGGCTCAT
CCTCCCCGCCCTGCGCATCTCTGGCAGCGCCCGCTCCAGGGAGATACTGTGATGCTTCAA

cDNA corresponding to *P. ananatis crtB* is on yellow background. Sequences corresponding to artificial ZYMV NIaPro cleavage sites are in blue.

>TEV-Ros1 (insert between possitions 8541-8542 of ZYMV-wt)

```
TCTGGCACAATGGAAAAGAATTGTCGTGGAGTGAGAAAAGGTACTTGGACCAAAGAAGAAGACACTCTCTTGAGG
CAATGTATAGAAGAGTATGGTGAAGGGAAATGGCATCAAGTTCCACACAGAGCAGGGTTGAACCGGTGTAGGAAG
AGTTGCAGGCTGAGGTGGTTGAATTATCTGAGGCCAAATATCAAAAGAGGTCGGTTTTCGAGAGATGAAGTGGAC
CTAATTGTGAGGCTTCATAAGCTGTTGGGTAACAAATGGTCGCTGATTGCTGGTAGAATTCCTGGAAGGACAGCT
AATGACGTGAAGAACTTTTGGAATACTCATGTGGGGAAGAATTTAGGCGAGGATGGAGAACGATGCCGGAAAAAT
GTTATGAACACAAAAACCATTAAGCTGACTAATATCGTAAGACCCCGAGCTCGGACCTTCACCGGATTGCACGTT
ACTTGGCCGAGAGAAGTCGGAAAAACCGATGAATTTTCAAATGTCCGGTTAACAACTGATGAGATTCCAGATTGT
GAGAAGCAAACGCAATTTTACAATGATGTTGCGTCGCCACAAGATGAAGTTGAAGACTGCATTCAGTGGTGGAGT
AAGTTGCTAGAAACAACGGAGGATGGGGAATTAGGAAACCTATTCGAGGAGGCCCAACAAATTGGAAATCAGGGA
GATACTGTGATGCTTCAA
```

cDNA corresponding to *A.majus Ros1* is on red background. Sequences corresponding to artificial ZYMV NIaPro cleavage sites are in blue.
